# Medico-Chirurgical Transactions, Published by the Royal Medical and Chirurgical Society of London, Vol. VI

**Published:** 1842-01-01

**Authors:** 


### Medico-Chirurgical Review,

### No. LXXI.

**[No. 31 of a Decennial Series.]**

OCTOBER 1, 1841, TO JANUARY 1, 1842.

MEDICO-CHIRURGICAL TRANSACTIONS, PUBLISHED BY THE ROYAL MEDICAL AND CHIRURGICAL SOCIETY OF LONDON. Second Series. Volume the Sixth. Octavo, pp. 253. Longman and Co., London, 1841.

THE present volume is as interesting as the general run of its predecessors. And this is no mean praise, for the Medico-Chirurgical Transactions occupy *the* very first place in medical periodical literature. The Profession in this country has reason to be proud of them, for a happier mixture of the abstract and the practical—of the curious and the useful, has never, we think, been published. It might be expected that, reflecting, as these Transactions do, the medical character of Britain, their general tone would be practical. And such is really the case. Not a volume but contains much that will help us at the bed-side, in diagnosis or in treatment.

The Table of Contents presents us with the following bill of fare;—

1. Observations on the structure of the entozoa belonging to the genus cysticercus, by George Gulliver, F.R.S. F.Z.S. Assistant-surgeon to the Royal Regiment of Horse Guards;—2. Case of osseous union of a fracture of the neck of the femur within the capsule, by Walter Jones, Esq., Surgeon, Worcester;—3. Observations on vaccination and small-pox, more especially with reference to the theory of vaccine influence, and the relations subsisting between the cicatrix and the character of the consecutive variola, by George Gregory, M.D. Fellow of the Royal College of Physicians, and Physician of the Small-pox and Vaccination Hospital, St. Pancras;—4. On gouty concretions, with a new method of treatment, by Alexander Ure, Esq. M.D. A.M. Member of the Royal College of Surgeons, London;—5. History of a remarkable case of phlebitis, with observations, by Thomas H. Silvester, M.D. Consulting Physician to the South London Dispensary, &c.;—6. Cases of cancerous or malignant disease of the spinal column, with remarks, by Cæsar Hawkins, Esq. Surgeon to St. George's Hospital;—7. A case of slow pulse with fainting fits, which first came on two years after an injury of the neck from a fall, with observations, by T. H. Holberton, Hampton, Surgeon Extraordinary to the Queen Dowager;—8. Fourth memoir on some principles of pathology in the nervous system, by Marshall Hall, M.D. F.R.S. L. & E.;—9. On dislocations, especially of the hip-joint, accompanied by elongation of the capsule and ligaments, by Edward Stanley, F.R.S. Surgeon to St. Bartholomew's Hospital;—10. Observations on the anatomy of the lungs, by Thomas Addison, M.D. Physician to Guy's Hospital;—11. Results of amputations at University College Hospital, London, statistically arranged, by John Phillips Potter, Esq. late House Surgeon,—with some remarks on the mode of amputation and method of dressing there adopted, by Robert Liston, Esq.;—12. Colica pictonum treated with warm water, by John Wilson, M.D. Physician to the Middlesex Hospital;—13. Case of malposition of the kidneys, absence of the vagina, uterus, and Fallopian tubes; disease of left ovary, by R. Boyd, M.D. Resident Physician to St. Marylebone Infirmary, and Lecturer on Medicine;—14. Pathological and surgical observations on the diseases of the ear, by Joseph Toynbee, Esq. Member of the Royal College of Surgeons, London, and late Assistant to the Conservators of the Museum of that Institution;—15. Two cases of dislocation of the tendon of the long head of the biceps humeri from its groove, by John Soden, Jun. Esq. Surgeon, Bath;—16. An account of two cases of aneurism of the superior mesenteric artery, in one of which jaundice was induced by pressure of the sac, by James Arthur Wilson, M.D. Physician to St. George's Hospital;—17. On congenital tumors of the pelvis, by Edward Stanley, F.R.S. Surgeon to St. Bartholomew's Hospital.

These articles we shall take in succession.

### I.—OBSERVATIONS ON THE STRUCTURE OF THE ENTOZOA BELONGING TO THE GENUS CYSTICERCUS. By *George Gulliver*.

We cannot present a full account of this paper, for which we refer our readers to the volume itself. The principal point in it is this. In a communication on certain oval corpuscles obtained from the genus cysticercus, read to the Zoological Society, by Mr. Gulliver, in March last, he drew attention to the fact that, if the white part near to the head of the entozoon be gently pressed, a little rather viscid fluid will escape, in which will be found a great number of oval corpuscles, presenting a beautiful microscopic object.

Mr. Gulliver's intention, now, is to shew the situation and extent which these bodies occupy in the worm, and their probable use in its generation; with an attempt to elucidate some other points in the structure of the parasite, hitherto imperfectly known.

Passing over the description of the oval corpuscles—of the bladder-like body, or caudal extremity of the worm and its containing cyst—and of the hooks or spines of the worm—we may notice Mr. Gulliver's hypothesis of the uses of the oval corpuscles. He thinks they are the ova of the cysticercus. " It will be difficult to entertain any other view of their nature when we recollect their heterogeneous structure, their regularity in size and shape, their aggregation together in the true body of the worm and the abundance of carbonate of lime contained in their shells. I am not aware that any gemmæ or sporules have yet been found to possess these characters; if, therefore, the oval corpuscles should be regarded as sporules, they must be sporules of a peculiar kind. As it is yet questionable how the corpuscles escape from the cyst, it will be necessary to institute a further examination of this part. I have not yet succeeded in detecting an aperture. But Dr. Knox has given an engraving of one instance in which the head and neck of the cysticercus cellulosæ was seen to project through a natural and well-defined opening in the cyst. With what facility the oval corpuscles may be disseminated will be understood, when it is recollected how prone they are to separate from the surface of the corium.

It is an interesting question whether the spherules of the caudal vesicle may not be considered as nuclei, which advance to the neck or body of the worm, and there become invested with cells, and so formed into the complete oval corpuscles.

Although the consideration of the basis of a methodical arrangement of the entozoa forms no part of the object of the present memoir, I may express my belief that an exact knowledge of the structure and arrangement of the claws of the cystoid family is likely to lead to an accurate discrimination of many genera and species hitherto but imperfectly characterized. Rudolphi, indeed, in the formation of his orders, availed himself of the characters afforded by the tentacles of the parasites; but although the importance of these parts was thus early appreciated, it does not appear to me that they have ever yet been described with sufficient accuracy.

It will be understood that the true body of the cysticercus is that part which, in compliance with custom, I have frequently called the neck; and that the globular portion of the hydatid is in reality the caudal or terminal vesicle."

### II.—Case of Osseous Union of a Fracture of the Neck of the Femur within the Capsule. By *Walter Jones*, Esq,

The case was that of a man, more than 80 years of age, who slipped down about the middle of October, 1838. He had the symptoms of fracture of the neck of the femur, and a long splint, on the outside, was employed. He died about a year and a half afterwards of disease of the lungs, having been able to move about with a stick, with the limb shortened about an inch and a half, and the foot much everted.

Mr. Stanley says, in reference to the appearances found after death:—"The history of the case is clearly that of fracture of the neck of the femur; the appearances of the bone show that there has been a fracture which has re-united by an osseous medium, and the direction of the fracture is such as, in my opinion, can permit no doubt that it was confined to the portion of the neck of the bone covered by synovial membrane; consequently, that it was wholly within the capsule. The fracture extends through the basis of the head of the bone in the line of its junction with the neck. As in other cases of the same kind, great part of the neck of the bone has disappeared, and, in consequence, the head is proportionately nearer to the trochanter major and shaft of the bone; its reunion has, in fact, taken place in part to the remaining portion of the neck, and in part to the shaft. This union is certainly osseous. In addition to the first maceration of the bone with its surrounding soft parts, it was subsequently immersed for several days in a strong solution of carbonate of potash, and one-half of the bone has been boiled in water for three hours without the slightest yielding perceptible in the line of the fracture.

The specimen is preserved in the Museum of St. Bartholomew's Hospital."

An interesting case.

III.—OBSERVATIONS ON VACCINATION AND SMALL-POX, MORE ESPECIALLY WITH REFERENCE TO THE THEORY OF VACCINE INFLUENCE, AND THE RELATION SUBSISTING BETWEEN THE CICATRIX AND THE CHARACTER OF THE CONSECUTIVE VARIOLA. By *George Gregory*, M.D.

Our able friend tells us :—

The epidemic of 1838 ceased with the frosts of Christmas; and from January 1839 to the end of September 1840, (a period of 21 months,) the metropolis was remarkably free from small-pox. The admissions into the Small-pox Hospital during the first three quarters of 1840 amounted only to 142, being at the rate of sixteen *per mensem*. In October 1840, a new epidemic began, and 46 patients were admitted in that month. In November the admissions were 64. In December, 75. From the 1st of January 1841, to the present day, (Monday, January 25,) the admissions have been 93, being nearly at the rate of 4 *per diem*—the greatest number ever admitted in one month since the establishment of the hospital in 1746.

Among the 327 patients admitted in 1840, 11 had complaints not proving to be of a variolous nature. Of the remaining 316, 194 were wholly unprotected, of whom 87 died, or 45 out of every hundred! 12[illegible] had previously been vaccinated, of whom 8 died, being in the ratio of [illegible] per cent. only. Two were supposed to have previously undergone small-pox.

Of the 316 patients, 47 were under 5 years of age, of whom 28 died; 45 were between 5 and 15 years of age (inclusive), of whom 9 died; 22[illegible] were adults, of whom 58 died. The total mortality was 95, or 30 per cent. on the gross admissions.

Dr. Gregory relates a case, and proceeds :—

"One hundred and twenty cases, I have said, of variola succeeding vaccination occurred at the hospital during the past year. My attention had long been directed to ascertain whether any, and what, relation subsisted between the number and character of the vaccine cicatrices, and the intensity of the consecutive variola; and I took this opportunity of investigating the subject. I found, b[illegible] careful inspection of the arms of those who attended for vaccination, and for re[illegible] vaccination, that much caution was requisite in forming any conclusion regarding the original vaccination, by the appearance of the arms in after life. It must always be borne in mind, that the full measure of vaccine influence is received by each individual on the eighth day from insertion. If the disease be subsequently rendered mild, either by art or peculiarity of habit, the cicatrix will [illegible] small and fugitive. Even should the vesicle be then destroyed by caustic, or t[illegible]

part cut out, and allowed to heal as a common sore, and no specific scar remain, still the individual would remain *vaccinated*. On the other hand, should the disease be aggravated by neglect or bad management, or a foul or inflammatory state of the child's blood, high secondary inflammation would be set up, and a large, wafery, or otherwise irregular cicatrix would form, which is permanent in after life. The amount of vaccine protection remains however in both instances *the same*, and the probable character of the consecutive variola receives no elucidation from inspection of the arm. The character of the cicatrix, then, depends more on the *accidental* or *secondary*, than it does on the *primary* or *specific* inflammation, and hence arises the small reliance which can be placed on it as a measure of vaccine protection.

Undoubtedly in the majority of cases wherein the cicatrices are *numerous*, *normal* and *well defined*, the consecutive variola is mild and varicelloid; again, where the consecutive disease proves severe, there the cicatrices will be imperfectly seen, or altogether wanting. But instances of the converse of these propositions are so numerous as scarcely to be called *exceptions* to a rule. As the profession are probably not so familiar with these latter cases, I have ventured to submit two opposing series to the notice of the Society. The first shows that small-pox after vaccination often proves *severe* where the cicatrices are *normal*. The second points out that the lightest and most truly varicelloid eruptions coexist with *small* and *very imperfect* cicatrices." 19.

The two cases which follow bear out the assertion, and shew that the cicatrix cannot be relied on as affording any certain test of the degree to which the constitution has imbibed an anti-variolous influence. Peculiarity of habit, similar to that which rendered certain persons patient of the variolous poison before the discovery of vaccination, and which now makes some patient under the venereal or mercurial poisons, and others irritable under the most minute quantities of those poisons, must probably be looked to as the best means of explaining the diversities in the aspect of consecutive variola. This is Dr. Gregory's opinion, and it seems very likely to be true.

Dr. G. next touches on the question, if the variolous and vaccine poisons are the same. After alluding to the affirmative conviction of Jenner, and the able experiments of Ceely, he sums up the evidence thus :—

"The morbid secretions from the cow, which possess the singular properties of transplantation to the human frame, of exciting there a like disease, and subsequently of protecting the human body, to a certain extent, from the assaults of small-pox, may be produced in that animal in four modes.

1. They are generated, spontaneously in the cow, under certain circumstances of soil, season, and locality. Such diseased secretions are often met with in cows soon after parturition, in the spring season, and when feeding upon young grass. But they arise also spontaneously from other and less known causes, and the disorder spreads like other epizootic maladies. It was this form of vaccine disease which Jenner chiefly studied.

2. The very same malady, developing the very same morbid secretion, is often observed to spread *by contagion*:—that is, by the application of the diseased secretion, thus generated, to the teats of healthy cows, differently circumstanced, by the hands of the milker.

3. The same morbid secretion, possessed of the same qualities, may be, and frequently has been generated in the teats of the cow by the application to them of the matter formed by the heel of the horse, when affected with the disorder called by farriers *The Grease*. This *greasy* matter may also be transplanted to man directly, without the intervention of the cow, proving that the anti-variolous property does not depend on any peculiar change which the virus undergoes while passing through that animal.

4. The same morbid secretion may be excited artificially in the cow by applying to the teats, or the mucous surfaces of the vagina, vaccine lymph from the arm of a child, even though 20 years had elapsed since that lymph had been humanized or assimilated to the human constitution.

5. To these four modes of exciting that kind of morbid secretion in the cow, which we call Vaccinia, (one constitutional and three artificial,) the labours of Mr. Ceely have now added a fifth. He has shown that the very same object may be obtained by applying to the mucous surfaces of the cow the matter of human small-pox. The vessels of the part are thereby excited to the production of a fluid or humour, identical in all its properties with that which arises from a constitutional and febrile disturbance in the cow's system, from contagion, from the matter of *Grease*, or the long-humanized vaccine virus.

"When we consider," adds Dr. Gregory, "that five modes of producing this morbid secretion in the cow are now known to exist, it is not unreasonable to suppose that others may hereafter be discovered. In this state of our knowledge, then, surely we cannot be justified in assuming the fifth and the last discovered of the whole, as the most important, and as affording the true clue to the mystery of vaccine protection. We should reflect that Mr. Ceely's experiments have entirely set aside Dr. Jenner's notion that vaccinia was the original or primitive poison, which time and fortuitous circumstances had aggravated into the malignant or secondary form, which we call small-pox. They have proved (if indeed they have *any* bearing on the intimate nature of these poisons) that small-pox is the *primary*, and cow-pox the *secondary* form. But when we further reflect on the absence of a contagious principle in Vaccinia, and the remarkable fact that febrile disturbance is not essential to its perfect development, we shall probably be nearer the truth in saying that the vaccine is a poison *sui generis;* that its relation to variola is still hypothetical; that the real and intimate nature of the protection which it affords is still unknown to us; and that a thorough acquaintance with its anti-variolous powers must be derived, not from analogy, but from an extended and careful observation of facts, continued through a long series of years.

It is worthy of record, that among the 120 cases of variola occurring at the hospital in 1840, subsequent to vaccination, eleven only were under 16 years of age. The youngest person admitted under such circumstances was of the age of 7. The first occasion on which I have ever known a child under 5 years admitted with small-pox after vaccination, occurred last week." 28.

Dr. Gregory adds the case. This, like all Dr. Gregory's communications on the subject is extremely valuable.

### IV.—ON GOUTY CONCRETIONS, WITH A NEW METHOD OF TREATMENT. By *Alexander Ure*, Esq. M.D.

Mr. Ure observes that it is well known that persons afflicted with gout are liable to the effusion of a white liquid into many of the internal cavities of the body. This liquid consists of serum and urate of soda, with sometimes a little urate of lime. In the course of time, the serous particles become absorbed, leaving a kind of soft clayey residuum, which afterwards becomes hard and friable; thus forming the so-called tophaceous concretions or chalk-stones.

Gout may be regarded as a specific inflammation which seems to affect the serous and fibrous structures. Accordingly, we find that the above depositions most generally take place in the cavities of joints, in the bursæ mucosæ, in the ligaments, neighbouring aponeuroses and cellular membrane, and in the periosteum. They have even sometimes been met with between the cutis and cuticle.

The effusion from which these concretions are derived, occurs not only during fits of gout, but likewise in the intervals; and as the extremities of the body, particularly the hands and feet, are the principal seat of the disease, it is there that the greatest accumulations take place. They occasionally, however, make their appearance about the articulations of the jaw and spine.

Although this process is usually preceded and accompanied by inflammation, yet there is no extravasation of coagulable lymph, no new covering or cyst surrounding the concrete matter,—like pus in an abscess. A circumstance which distinctly proves that the inflammation is not of a phlegmonous character, and that the non-absorption of the deposit is to be ascribed to physical causes.

Gouty tophus is not confined to man; it occurs also, though more rarely, in animals, when placed in a similar condition, that is, when fed for a length of time upon a highly azotised diet. Aldrovandi has detailed its effects in birds of prey, more especially in hawks. Gout-stones sometimes attain a very great size. Now, says Mr. Ure,

"Since one part of urate of soda requires about 4000 parts of water to dissolve it, it may be reasonably assumed that the refractory nature of the above deposits is due to their very sparing solubility in the fluids with which they come in contact. It therefore occurred to me as a consequence deduced from some researches into the composition of the renal secretion in certain of the lower animals, that some means might be devised to enable us through the medium of the circulation so to modify that secretion in man as to supersede, for a time, the urates altogether.

The graminivorous animals, as the horse and cow, secrete from the kidneys a peculiar acid (the hippuric). It is present in their urine combined with soda. Now, the hippurate of soda, which may be considered the analogue of the basis of gout-stones, is an exceedingly soluble salt, (requiring only two parts of water, at 60° F., to dissolve one), as are likewise the hippurates of potash, of ammonia, and of lime. Hence it appeared probable that were we to adopt such therapeutic measures as would determine the human kidney to secrete this acid instead of the uric, we should thereby, in all likelihood, controul and prevent the deposition in question.

I ascertained in the course of last summer, by repeated experiments, made first of all upon myself, and afterwards upon individuals labouring under gout, that the above substitution could be perfectly accomplished without the slightest risk of affecting the general health, or of irritating the urinary organs. The substance employed for this purpose was the benzoic acid. If an hour after a meal, a scruple of this acid be taken into the stomach, in the course of a couple of hours subsequently the urine voided, amounting to five or six ounces, will be found, on adding a small quantity of muriatic acid, to yield a copious precipitate of beautiful rose-pink acicular crystals, which weigh, after being allowed to settle for a day, about fifteen grains. This quantity is by atomic computation equivalent to little more than one-half of the benzoic acid expended, so that the remainder must have made its escape by some other emunctory, probably the skin.

The above crystals, when examined by the microscope, display the characteristic form of the hippuric acid, namely, a four-sided prism, with a dihedral summit."

"It may be observed, that no trace whatever of uric acid, or of any of its salts, or of benzoic acid, could be discovered in the above urine.

A nearly analogous result is obtained when benzoate of ammonia or of potash is administered; and, under particular circumstances, the exhibition of one or other will be found preferable to the simple acid; either in the neutral state, or with an excess of base, when there is a disposition to acescence in the *primæ viæ;* apportioning the dose, in every instance, to the condition of the urinary secretion, previously ascertained by analysis.

By this singular interchange of elements, capable of being effected only by the aid of vital chemistry, we have an organic acid, containing 8 atoms of azote and 10 of carbon, replaced by one containing no less than 18 of carbon and only 2 of azote, and that even in what various eminent pathologists regard as a highly azotised state of the system.

It is obvious that this new plan of treatment, and which does not interfere with other remedial means, must be steadily persevered in for a considerable length of time, ere any adequate benefit can ensue. How far it may be applicable to various forms of calculous disease, connected with the gouty diathesis, remains for future investigation to decide. Most unequivocal proofs have already been afforded me, of its efficacy in correcting and removing certain disordered states of the urine in individuals prone to attacks of gravel." 35.

### V.—History of a Case of Phlebitis, with Observations. By *Thomas H. Silvester*, M.D.

This is really a remarkable case. We hardly see the possibility of condensing the excellent account of it furnished by Dr. Silvester.

*Case.*—Mr. P., æt. 59, observed on Friday evening, March 27th, a pimple on his upper lip, which he supposed to have arisen from a scratch in shaving. His friends had noticed him carrying his pen, whilst engaged in accounts, transversely between the lips. He had been using a steel pen, and a new metallic ink, and it appeared to them that this fluid had accidentally come in contact with the wound, and imparted to it the peculiar deep-red irritable aspect which was very observable. On the following Tuesday the patient retired to bed early, complaining of general uneasiness, but neither fever nor head-ache were present, and he slept well. Dr. S. saw him, for the first time, the next day, (Wednesday,) his pulse was 120, small and weak, the skin cool, and he did not complain of thirst. The countenance was inexpressibly anxious, not unlike that of a person who has taken poison, or one who has been stabbed in the abdomen; the lip was greatly swollen, and he suffered more from a feeling of distention than from pain. So much difficulty was experienced in the attempt to open the mouth, that it became next to impossible to ascertain the state of the tongue; another hindrance arose from the tumified overhanging lip, beyond which the tongue could not have been projected had no other impediment existed. The tumefaction extended a little way upwards on each side of the alæ nasi, but not at all downwards towards the lower lip; it was of a dark red, almost livid, hue, and very firm to the touch. There were no vesicles nor bullæ, nor œdematous appearances on pressure; the pain was of a distending, and not of a burning kind. The affection so resembled the asthenic form of erysipelas, that Dr. Silvester determined upon tonic treatment. The patient was desired to take from four to six ounces of port wine in boiled sago during the twenty-four hours, and three grains of the disulphate of quinine every four hours. This mode of treatment seemed to promise the best effects,—the pulse became fuller and stronger, and the swelling ceased to spread,—the lip itself was cool, but of an immense size,—a glutinous exudation, which now appeared on its surface, thickening gradually from day to day by fresh additions, assumed at length the appearance of a coarse scab, with rocky projections, so perfectly inflexible, that not even the slightest movement of the lip could be effected.

The gums, and the interior of the mouth generally, were seen to be of a dark livid hue, and viscid saliva flowed unceasingly over the neighbouring parts, occasioning soreness and excoriation. On the fourteenth day of the disease, the skin was cool and perspirable, the pulse 80, still rather feeble, the lip was nearly free from uneasiness, although much swollen and thickly encrusted: the patient had slept well the previous night, he was enjoying, with a good appetite, his sago, and believed himself to be rapidly recovering. Dr. S. observed on the very next day a large red cord, apparently terminating in a vein extending upwards at the side of the nose as far as the inner angle of the eye, first on the left, and afterwards on the right cheek. These inflamed vessels greatly resembled irritated absorbents, but they were of much larger size than the latter, being as big as a goose-quill, even whilst no fluid could be detected in their interior by the touch. After the lapse of five or six days, fluctuation was distinctly perceptible, and Dr. S. then noticed at several points in the course of these suppurated veins, a slight degree of redness. These red spots became the seat, each of an exudation precisely of the same character and appearance as that which had previously been observed on the upper lip. A viscid liquid at first escaped, and this, either hardening, or being succeeded by a secretion of thicker consistence, a series of projecting masses, somewhat larger than a horse-bean, of a yellowish green hue, not unlike the scabs of rupia, appeared along the course of the vessels. On one of these scabs or exudations being loosened from its seat, at a subsequent period, pus continued to flow for several days from the part which had received the violence. Another, which had escaped injury, and maintained its position up to a period within one week of the patient's death, dislodged itself spontaneously from the depression between the eyebrows, which situation it had occupied, leaving the parts underneath perfectly sound and healthy, but rather redder than natural.

On the twentieth day of the disease the veins of the forehead had begun to swell, and in a very short period they were to be seen, in great numbers, ramifying all over the fore part of the head, and extending beyond the vertex, presenting a most extraordinary appearance, difficult for the pencil to portray. The skin and cellular membrane occupying the spaces between the several inflamed vessels seemed at this stage of the complaint to be almost wholly unaffected, and owing to this circumstance, the veins themselves were highly prominent and easy to be distinguished from any other order of vessels. A process similar to that which had taken place in the veins of the lip and face, occurred in those of the scalp, namely, exudation of a glutinous fluid, and incrustation in some instances—resolution and suppuration in others.

The incrustations became loose invariably on the application of a poultice, and quickly fell off. The interior of the vein was thus exposed, and a long, irregular, ulcerated cavity formed.

The vessels which were opened with a lancet emptied themselves gradually of their contents—pure laudable pus for the most part—and neither exudation of the thin gelatinous fluid nor ulceration took place.

On the 21st of April, skin cool, pulse 82, feeble but regular. 24th—the pulse had suddenly risen to 100, extremely feeble.

On the 6th of May the patient seemed to have had a shivering ; it was however very slight : he had likewise vomited after his usual night dose of Syr. Papav. 8th—greatly excited after a dose of morph. hydrochloras gr. ss. 26th—the patient expired, perfectly rational to the last moment.

*Autopsy.*—The body greatly emaciated ; on turning back the scalp, which was so fragile and perforated by ulceration that it tore, and yielded to the slightest touch, the diseased veins were seen, meandering over the internal surface, filled in some part of their course with a yellowish crumbling, fibrinous mass, the smaller branches containing fluid blood of a pale colour, in minute quantity, and a single trunk of the temporal on the left side terminated in a foyer filled with laudable pus. On slitting open those veins which, during life, appeared restored, or nearly so, to their natural condition, they were found perfectly empty, rough and irregular in their interior, apparently deprived of their smooth lining, and their calibre greatly increased. The shrunken attenuated muscles were cut into and examined in several parts of the body, but no deposits of pus could be detected. The lungs, liver, kidneys, and brain were in a healthy condition, but bloodless. The heart and larger vessels entirely empty. The joints were not examined internally, but they evidently contained no fluid. The patient had not complained of pain or uneasiness in the limbs, and had even walked across the room a few days before his death.

Dr. Silvester makes some judicious reflections on the case, which presents many features of singularity. Unfortunately, there seems to be no opportunity of drawing from it any great practical lesson.

### VI.—CASES OF CANCEROUS OR MALIGNANT DISEASE OF THE SPINAL COLUMN, WITH REMARKS. By *Cæsar Hawkins*, Esq.

Mr. Hawkins observes that there are so few well-described examples of malignant disease of the spinal column, that he is induced to relate four cases which have come under his observation. We shall endeavour to abbreviate them.

*Case* 1.—Sophia Green, aged 39, admitted into St. George's Hospital,

May 7, 1834, with ulcerated cancer of one breast, thickened skin, and enlarged absorbent glands. She complained however quite as much of pain in the neck, which had begun about two months previously, the pain being in the cervical vertebræ chiefly, whence it extended round the neck and over the scalp, and all these parts were very tender to the touch. She had also a little difficulty and pain in swallowing, and was unable to move her head in any manner without great exertion, and the effort gave her considerable pain. Supporting her head and neck on the right side, as she lay in bed, was very painful, but she could not lie on the left side at all; probably some stretching of the parts being occasioned by the weight of her head on the pillow. If she tried to change her position, it was done by first putting both her hands to her head to support it; and pressing the head downwards by the hand occasioned much suffering. The centre of the neck appeared a little sunk forwards, as if the upper vertebræ had been depressed in that position. In the early part of June an immense number of cancerous tubercles formed in the skin around the breast, covering much of the abdomen, and thorax, and shoulder; and in the latter situation many of them had coalesced, so as to form a hard mass of considerable size. About the same time she began to suffer from obstinate constipation, with frequent vomiting—the breast sloughed extensively—and she died on the 16th of July.

*Dissection.*—The breast and pectoral muscles, and cellular texture, formed a large mass of scirrhous tumor, which at one part reached to the intercostal muscles, and a central slough had contaminated the ribs also, two of which were softened by absorption of their earthy substance, and filling of the cells with bloody pulp, so as easily to allow of their being bent or cut. The cutaneous tubercles, and absorbent glands, and the fat surrounding them, were evidently of cancerous structure, and one of the glands contained some bloody purulent fluid. The body of the fifth cervical vertebra was very irregular on its surface, and was softened throughout with much enlargement of the cells of the cancelli, which were filled with a sanguineous pulpy fluid; the two adjoining vertebræ showed a lesser degree of the same morbid structure. The uterus was healthy, but both Fallopian tubes were much dilated, and contained a thick brownish fluid; the ovaria formed two tumors of the size of an orange, and contained several cysts, the larger of which were filled by transparent fluid, the others by an opake semi-fluid substance. These ovarian tumors were the immediate cause of death, as they filled the pelvis in such a manner as to obstruct the rectum, which lay in the angle between them, upon the sacrum; and their nature was probably malignant, as several of the lumbar glands were enlarged and pulpy.

In this case the cancerous change of structure was in an early stage, affecting only the cancellous texture, or nearly so, and influencing slightly the adjacent nerves; but not forming any tumor, and causing no alteration in the functions of the spinal marrow. The next case illustrates the further progress of the disease.

*Case* 2.—Jane Hall, aged 55, admitted for paraplegia under Dr. Wilson, transferred to Mr. Hawkins, in November, 1839. The right breast had been removed six years previously for cancer, and the part had remained well till May of the present year, at which time some cancerous tubercles were formed in and around the cicatrix, and one or two glands became enlarged and hard in the axilla.

About March she had begun to suffer much pain in the back, chiefly in the dorsal region, and in May she experienced some pricking sensations in the feet, soon followed by numbness and loss of sensation, and in a short time by loss of power over the muscles of the lower limbs, which nearly at the same time became affected by involuntary contractions; in July the bladder and rectum also became paralysed.

In November, one spinous process in the back was just enough prominent to be fixed upon as the seat of the disease, but by degrees it projected so as to form an acute angle, with a considerable curve above and below it, the vertebræ being bowed forward somewhat in a semicircular form. She complained of constant and acute pain along nearly the whole spine, but especially at the angle formed by the sixth dorsal vertebra, and the pain was much increased by pressure about this part, or any where below it, and for a little way above that bone. The angle appeared to arise from loss of substance in the body of the vertebræ, without any apparent swelling around it. Below the affected part of the spine all the functions of the spinal marrow were materially impaired. And Mr. Hawkins gives a very interesting account of experiments, shewing the absence of voluntary motion with the persistence of the excito-motory power, &c. Sloughs formed on the trochanters and sacrum, and on June 17, 1839, the patient died.

*Dissection.*—All the vertebræ were found to be unusually soft and vascular, and there were seen in the section of several of the dorsal vertebræ spots or tubercles of yellowish white substance, similar to that found in larger quantities in their bodies. On opening the sheath of the spinal marrow, a little clear water was seen below the arachnoid membrane, but the whole medulla was of its natural colour and consistence, and presented no appearance whatever of inflammation. Opposite to the sixth dorsal vertebra it had been pressed upon by a tumor projecting from the body of the bone, so that a deep sulcus of the entire circle was formed, in which so little medullary matter was left that the central part was almost transparent: the part thus pressed upon was full half an inch in length. Even in this point, however, there appeared to have been no inflammation whatever.

Opposite to this part some firm structure without bone projected from the body of the sixth dorsal vertebra in the form of four oval prominences, so as to encroach considerably on the canal; three of these were covered by the dura mater in firm union with the morbid growth, but over the other an opening was formed in the membrane with smooth edges, through which the new growth appeared, the opening being the result of simple absorption without any ulceration. On making the section of the vertebræ seen in the preparation, the morbid growth was found to proceed from these vertebræ, the sixth or central one being most altered in shape, though all three of their bodies were almost entirely converted into a cancerous substance; the sixth having spread out posteriorly, while it was much compressed in front, the fifth and seventh vertebræ almost coming in contact, an acute angle was thus produced, which made the spinous process of the sixth so prominent during life. The projection into the canal was about half an inch beyond the line of those vertebræ which had no external growth; in several of the other vertebræ, however, cancerous substance had been deposited in the form of tubercles in their cancelli. The new growth was firm, with fibrous structure of a white appearance in bands, with some yellow softer substance in the interstices. Many of the other bones of the body, and the other parts of those in which the cancerous tubercles were seen, were softer and more vascular, and with larger cells than usual, with reddish pulp in their cancelli.

The lungs were healthy, but the pleuræ were every where closely adherent, so as to form a thick layer of very hard substance, which had more the appearance of cancerous membranes than of simply inflamed pleuræ.

The abdomen contained a small quantity of serum, and almost the whole of the peritoneum was covered by small cancerous tubercles, in no part going into any viscus: they were hard and close set, of the size of grains of wheat, but a few were a little larger. They were most numerous on the diaphragm and small intestines, and many parts of the bowels were matted together by adhesion of these tubercles, especially about the head of the colon and the ileon. The omentum was changed into a hard scirrhous band about an inch broad, and half an inch thick.

The peritoneal surface of the uterus and adjoining parts was much altered by the tubercular deposit, and in the body of the uterus was embedded a small cancerous tumor the size of a pea.

The axillary glands were of well-marked scirrhous character, with bands going far into the surrounding fat; the cancerous cicatrix and cutaneous tubercles did not affect the muscles below them.

After alluding to two cases of Sir Astley Cooper's, and one of Sir Benjamin Brodie's, Mr. Hawkins remarks:—

"As a diagnostic sign of cancer of the spine, acute pain cannot therefore be regarded as *invariably* present, but in all the other four cases it appears to have been most excruciating; I never saw evidence in any other disease of the spine of such exquisite suffering as in my two cases; and as pains have generally been noticed as having preceded fracture of the femur or other bones, when affected with cancer, the practical inference may perhaps be legitimately drawn, that rheumatic pains much complained of in the spine, should usually contra-indicate the performance of an operation for cancer of the breast or other organ. In Green's case the acuteness of the pain and tenderness, and the manner in which certain nerves of the head, throat and neck were affected, enabled me some months before her death to anticipate cancer in the vertebræ; and in Hall's case also, the same circumstances, together with the degree, and in some measure the peculiar manner in which the functions of the spinal marrow were interfered with, left no doubt in my mind of there being cancer, although the angular curve made the local appearance not unlike that of caries." 59.

The preceding cases were instances of scirrhus of the spine succeeding scirrhus of the breast. The next case is adduced as one of primary scirrhus of the spine itself.

*Case* 3.—A gentleman, aged 74, after sitting near an open window in the Spring of 1840, began to feel pain on the left side of the neck, extending from the head to the shoulder, with numbness of the left side of the head. The pain was like that of rheumatism, and never subsided after its first appearance, and in about a month was succeeded by a swelling on the left side of the neck. Both pain and swelling increased, and in July, about four months from its commencement, the pain became more severe and burning, and both pain and swelling extended to the right side of the neck. When he came to town at the end of August, the pain was most severe on the right side, where a spot of the size of half-a-crown, about the middle of the neck, near the vertebræ, was affected with most excruciating pain; the pain every where was severe, but he said he could bear the rest tolerably well, if this part was relieved. Mercury and iodide of potassium, with leeches and cold lotions effected some diminution both of the swelling and the pain. In about three weeks Mr. Hawkins saw him.

In the back of the neck was a good deal of swelling, of a firm elastic character, appearing to be below the muscles, but now and then giving an obscure sense of deep fluctuation; the swelling on the left side of the spine reached from the head to the level of the fifth cervical vertebra, and on the right was opposite only to the second and third bones, projecting chiefly at the sides, so as to leave the sulcus of the spinous processes in some measure perceptible. This swelling was very tender, as well as painful, without adhesion to the skin, or alteration of its colour. Every attempt to move occasioned very great suffering, so that it was difficult for him to find a tolerably comfortable position for the head to lie on a pillow; no movement whatever was performed, but by the head with the whole body, and any attempt forcibly to rotate the neck could not be borne for an instant; pressure on the head downwards produed also much increase of suffering. The pain was constant, but liable to occasional increase, it prevented sleep, and required the free use of opiates. The tumor diminished, but afterwards increased again. About three weeks before his death, his left arm became nearly paralysed, and the power over the left leg was slightly impaired, his senses being the whole time perfect.

During most of the time the general health was little disturbed, but latterly he suffered a good deal from salivation, and became thinner and weaker, and thought himself in danger, and during the last three weeks, in the last of which he had severe diarrhœa, his strength gradually failed.

*Dissection, Oct. 25th.*—The swelling was composed of a firm solid tumour, which occupied the place of the third cervical vertebra, and in part the second also, with a considerable portion of the adjacent ligamentous, tendinous and muscular substance, the distinction between what had been of osseous, and what of soft structure, not being clearly perceptible. The new structure occupied the whole of the arches and processes of the vertebræ, and in part their bodies also, so that scarcely any part remained osseous except the processus dentatus, which was by the softening of its base so moveable, that there must have been some risk during life, of its being torn away from its attachment. Some of the new structure had encroached on the vertebral canal on the left side, between the dura mater and the first and fourth vertebræ, the tumor adhering slightly to the membrane; and within it was a good deal of thin serum, but the medulla itself was not unusually vascular. The brain also was healthy, with the exception of some serum effused under the arachnoid of the cerebrum.

The tumor was white and lardaceous in appearance, and softest in the centre, where the bone originally existed, which part was also more vascular than the outer part, which had been formed by the softer tissue around the vertebræ; on the left side, where the natural texture of the muscles began to be evident in union with the morbid growth, was a small quantity of dark, bloody pus. In the liver, which was otherwise healthy, were two tubercles of the same white texture as the tumor, but a little firmer, and less lardaceous; one of these was on the surface, and of the size of a walnut; the other was in the interior, and a little smaller. The kidneys and other viscera, both of the abdomen and thorax, were healthy, except that the intestines were blanched by the diarrhœa.

*Case* 4.—C. Gibson, aged 4, admitted into St. George's Hospital, October 30, 1839, with the left nostril distended with a malignant tumor. Sir B. Brodie removed this, destroying the surface to which it was attached by means of the chloride of zinc. The child left the hospital at the end of January, there appearing to be only some dead bone to exfoliate. On Feb. 19, however, he was re-admitted, looking pale and anxious, and scarcely able to move any of his limbs, in which much pain was felt. The abdomen was large and tender, there was a good deal of fever, and the nostril was full of tenacious mucus, with some coagulated blood.

After his admission, the paraplegia increased, as to the muscular power, but the sensibility was not lessened, nor was there any sloughing of the lower part of the body; and somewhat later there was incontinence of urine and fæces.

Some bleeding continued to take place from the nose, with sloughing of the cheek, by which a large part of the maxillary bone was exposed, and its vitality destroyed, and in March, some glands behind the left ear enlarged considerably, and a tumor was felt at the extremity of the sternum. The general strength gradually declined, with much irritation from the gangrene of the face, and the child finally sunk on the 12th May 1840, but without any loss of mental power.

The parts about the head had undergone much alteration; the maxillary bone was softened in the interior, and the antrum filled with soft medullary matter; the æthmoid and sphænoid bones were similarly changed, as well as the dura mater lining them, and the cells were obliterated by the morbid structure. Some new growth occupied the sphænoid fossa, and a portion coming into contact with the periosteum under the zygoma, the disease of the outer membrane had produced a hole through the temporal plate of the sphænoid bone, so as to project through it into the cranial cavity. The brain itself, however, was healthy.

The tumor at the end of the sternum had been formed all round the ensiform cartilage and adjacent bone, the cartilage itself being unchanged in the midst of a solid semi-cartilaginous mass, of the size of a large walnut, and of a yellowish white colour. A great many similar tumors existed in many other parts of the bones, which were almost every where confined to the periosteum, so that they could generally be easily separated from the bones by tearing off their investing membrane; the largest of these was on the inside of one ilium, and was three or four inches in diameter. They were most numerous along the front of the vertebræ, and on the ribs; and sections of the dorsal vertebræ showed that in several of these bones the new structure had spread into the cancellated texture, the outer shell being absorbed; and in the osseous tissue, as in the cells of the nose, the morbid growth was diffused and softened, so as to resemble medullary tumor, while all those of the outer part of the bones or cartilages were firm, and like fibrous cartilage in appearance; the intervertebral substances were wholly unchanged. Much new growth had also spread between all the processes of the vertebræ, and several masses of some size were formed on the posterior part, and, in one section, the dura mater was thickened by new growth, but smooth on its inner surface. The appearance of the medulla spinalis in its recent state was unfortunately not observed; as far as can be observed, however, in its present condition, when hardened in spirit, it seems to have been irregularly pressed upon by the morbid growth within the spinal canal, but not to be otherwise altered in texture.

The vertebræ most affected were those of the back, which have been preserved; and from the tumors in front of these bones, a great mass of similar hard or cartilaginous appearance, and apparently of globular portions united, projected forwards in the centre of the chest, whence it extended into the root of each lung, the texture of which was thus mixed with divided portions of the general mass; and in some parts towards the circumference of the lungs, and under the pleuræ, were separate tubercles of similar hardness and appearance to those in the periosteum. No tubercles were observed in any of the other viscera; but the number existing on all the bony parietes of the abdomen accounted for the fullness and tenderness of that part during life.

These cases of Mr. Hawkins' will be perused with interest, in the dearth of well-described instances of medullary disease or scirrhus of the spine.

### VII.—A Case of Slow Pulse with Fainting Fits, which first came on two years after an Injury of the Neck from a Fall. With Observations. By *T. H. Holberton,* Hampton.

A gentleman, aged sixty-four, in December 1834, whilst hunting, fell from his horse on his head, with his chin thrown violently on his sternum. He was stunned, and on recovering himself, he said that he "had broken his neck." He complained of general soreness and stiffness, and of great pain in the neck about the cuneiform process and condyles of the os occipitis. He was helpless and could not move in bed, and was totally unable to rotate the head. He was cupped, &c., and the head supported by an air-collar. The pain in the neck continued about six weeks. A year afterwards he looked well, but had still a difficulty in moving his head.

There was no further particular observed in this case until January 1837, when he had a fainting fit, whilst walking out. Mr. Jackson of Stamford was sent for, and he found the pulse at twenty in the minute. He had another attack, after excitement, in the same year, and another in the following June.

In March, 1837, Mr. Holberton first saw the patient. Then "his pulse when he was free from excitement or casual disorder, was thirty-three, but it was easily altered. Mental excitement usually increased it, and, in general, this was followed by a corresponding slowness of the pulse, and often by a fainting fit; and a sudden rise of the pulse, or even a gradual increase above the point, that might in his state be called his healthful standard, usually indicated mischief, and was found to be a bad symptom. Costiveness, and disorder of the stomach and bowels, always affected the pulse, by increasing or diminishing it, and were the most invariably exciting causes of a fainting fit. Gout, to which he was very subject, was another cause.

The general character of the pulse, when he felt well, and was free from disorder, was firm, full, and free; sometimes quite regular, sometimes intermittent.

The attacks increased in frequency as well as in degree, as time advanced, and the first most severe and alarming *succession of fits* occurred in June 1838. On the previous day, this gentleman had eaten heartily of a variety of substances at his dinner, and on the following day about one o'clock P.M., syncope came on, and a succession of fits continued till half-past six or seven in the evening, with intervals of one or two to fifteen minutes between the attacks. I gave him brandy and other stimuli, without stopping or even abating the fits; on the contrary they seemed to increase the mischief, for they made him sick, and disordered his stomach. His pulse on this occasion sunk considerably; it chiefly ranged between twenty and fifteen per minute, but at times it fell to twelve, ten, nine, eight, and at three or four different times when the patient was quite sensible and not in a fit, I counted his pulse as low as seven and a half in the minute. Dr. Mitchell on a subsequent occasion also observed this very low state of pulse, as did Mr. Cullen, who was then acting as my assistant.

If the finger were placed on the radial artery, the approach of a fainting fit might always be known, sometimes for a second or two before it manifested itself by any change of the countenance. The pulse would cease before the syncope took place; and the fit would continue till the heart again beat, when the face would redden and consciousness return with a wild stare and occasionally a snorting, a slight foaming at the mouth, and a convulsive action of the muscles of the mouth and face.

The frequency of the attacks was uncertain. Sometimes the patient would have two or three in a day, sometimes one in two or three days, at other times one in a week: sometimes one in a fortnight, or three or four weeks. Sometimes the fit would be severe and all consciousness be lost, at other times there would be a mere threatening or giddiness."

The treatment that agreed best consisted in carefully regulating the bowels, preventing the formation of acid in the stomach, giving a plain nutritious diet, with three or four glasses of wine a-day, or a proportionate quantity of brandy and water. The best plan during a fit, was simply to fan the face, apply Eau de Cologne, &c., to the nostrils, forehead, and temples, and, if there were a disposition to a continuance, to give coffee or tea.

His final and fatal attack occurred in April, 1840, at dinner.

*Dissection.*—The heart was large, and there was some thickening of the lining membrane, with some increase of size in the left, and more in the right auriculo-ventricular opening.

The dura mater was very firmly united throughout its whole extent to the cranium, which was dense and unusually thin. There was a large quantity of serum contained in the cavity of the arachnoid. The substance of the brain was slightly congested. It was in other respects perfectly healthy. The medulla oblongata was small in size and extremely firm in consistence. The foramen magnum was altered in shape. The antero-posterior diameter much diminished. The superior part of the odontoid process of the axis appeared to have been pushed back, and somewhat raised above its usual situation. The antero-posterior diameter was so much narrowed that it would not admit of the little finger. The dura mater and ligament covering the posterior part of the body of the axis, were very much thickened. The atlas was in its usual situation, but the articular cavities were firmly ossified to the condyles of the occipital bone, and permitted no motion whatever between the atlas and skull. There was a slight unnatural projection on the lamina on the right side, between the spinous process and articular process of the axis.

Mr. Holberton, in some observations, gives the following rationale of the case. The injury to the occiput and to the first and second vertebræ at the time of the fall, must have been very great, though insufficient then to cause any visible effect on the functions of the spinal chord.

Inflammation, however, followed, and a consequent thickening of the ligaments, which narrowed the foramen magnum and upper part of the spinal canal, and thus affected the medulla oblongata and upper part of the spinal chord, diminishing the size and increasing the density of these parts.

### VIII.—MEMOIRS ON SOME PRINCIPLES OF PATHOLOGY IN THE NERVOUS SYSTEM. By *Marshall Hall,* M.D.

This is the *fourth* Memoir contributed by Dr. Hall to the Medico-Chirurgical Transactions. It is,—

### ON THE PLAN OF OBSERVATION OF DISEASES OF THE NERVOUS SYSTEM.

Dr. Hall remarks that sufficient has now been done to shew that we, in all investigations of the nervous system, view it as subdivided, not into the cerebro-spinal, and the ganglionic; but into the cerebral, the true-spinal, and the ganglionic; and that, in considering each disease of the nervous system, we must trace its influence distinctly in these three sub-divisions of that system; or, to state this view more emphatically, we must inquire,—

1. *What are the distinct diseases of the cerebral, of the true-spinal, and of the ganglionic sub-divisions of the nervous system?*

2. *What is the influence of disease of one of these systems on the other two respectively?*

3. *In what order is that influence manifested?*

Besides these important questions, there are several others not less momentous. They are these,—

1. *What are the effects of irritation and of counter-irritation, of pressure and of counter-pressure, in diseases within the cranium or the spinal canal?*

2. *What is the special anatomy of the* base *of the encephalon, and its relation to cerebral diseases?*

3. *Why, with* similar *symptoms, have we* dissimilar *morbid appearances within the cranium; and vice versâ?*

4. *What are the diseases of the nervous system in which we find, generally speaking,* no *morbid appearances on a post-mortem examination?*

Dr. Hall proceeds to treat briefly these questions and subjects in succession.

1. *What are the distinct Diseases of the Cerebral, the True-spinal, and the Ganglionic Systems; their mutual influence, and the order in which they are manifested?*

Dr. Hall remarks,—If disease be limited to the cerebrum, its influence is limited to the cerebral functions. If from the cerebrum it extends its influence to the true spinal marrow, the functions of this latter are involved, and spinal symptoms are added to the cerebral; or with the cerebral the spinal functions are impaired. This latter condition may frequently be detected by using the reflex function as a *test;* and in this manner the views of this function, which have recently been laid before the profession and before this Society, come to have their practical application. They afford, indeed, a new source of diagnosis of the nature, seat, and extent of diseases of the nervous system, and consequently of their prognosis.

If the disease be limited in its effects to the true spinal marrow, the symptoms are exclusively spinal, sometimes in excess, sometimes in a defective form. If the cerebral system become also involved, cerebral symptoms are superadded to the spinal.

Similar remarks may doubtless be made in regard to the ganglionic system, viewed in its connections and relations with the cerebral and spinal.

He cites hemiplegia as an instance of affection of the cerebral, tetanus as a sample of that of the true-spinal functions.

*Hemiplegia.*—In cases of hemiplegia, observes Dr. Hall, the *danger* is precisely in proportion as spinal symptoms are superadded to those of the cerebral system. If the respiration be stertorous, if the deglutition be difficult, if the functions of the bladder, rectum, and sphincters be impaired, there is great danger; if these events *continue* for a considerable time, or if they *supervene,* the event is always fatal.

The spinal symptoms which exist at first, and gradually yield, probably depend on *counter-pressure* from congestion; this counter-pressure is relieved by blood-letting, &c., and its effects cease. When, on the contrary, the spinal symptoms continue, in spite of the remedies, they probably depend on the extent of the effusion; and this cannot be remedied.

*Apoplexy.*—"I need scarcely remark, that in congestion of the cerebrum, in apoplexy, as well as in hemiplegia, if the symptoms of affection of the true-spinal system *continue,* the issue is fatal. In such cases the patient dies of asphyxia; and I cannot but think that tracheotomy might sometimes allow time for the operation of remedies or of nature's resources, and prevent a fatal result. It is well known to the members of this Society, that this operation, performed by Mr. Sampson, of Salisbury, saved the life of a poor patient, dying from the apoplexy of deep intoxication.

I may here observe, that if the stupor and stertor continue, the next series of phenomena are those observed to result from defect of the function of the ganglionic system. The bronchi become clogged with mucus, and the intestines distended and tympanitic from flatus. M. Andral observes—'Le stertor de la respiration est en géneral un signe d'un très fâcheux augure, et il est rare que les individus qui le présentent d'une manière prononcée échappent à une mort prochaine. Pour l'expliquer, on trouve sur le cadavre un engoument considerable du poumon, et beaucoup de mucosités spumeuses dans les bronches. C'est véritablement par la gêne de la respiration que succombent les sujets frappeés d'hémorrhagie cérébrale, dans le cas où l'attaque est forte, et où ils meurent promptement.' It is obvious that the stertor is *not* explained in this manner; but that the bronchial and tracheal rattles which occur under these circumstances *are* so explained. They constitute, in effect, two orders of phenomena. The stertor depends on affection of the medulla oblongata; the crepitus or rattle on that of the ganglionic system. The latter is precisely the effect observed by Sir Benjamin Brodie, Sir Astley Cooper, and other physiologists, in animals in which the pneumogastric nerves had been divided. But the stertor depends upon the affection of the true-spinal system." 92.

*Hydrocephaloid Disease.*—"I attended the son of Mr. Howlett, in Thayer-street, in consultation with Mr. Grant. The little patient was four years old, and laboured under symptoms which seemed to denote the existence of hydrocephalus; there was a state of stupor; the eye-lids were only partially closed, and they were immoveable on the approach, and actual contact, of the finger; the respiration was irregular, and the pulse frequent. I observed that the phenomena presented by the eye-lids would afford a criterion, which would suggest both the diagnosis and prognosis. The history, and the cool and pale condition of the cheeks, suggested the hope that the symptoms depended more upon exhaustion than actual disease within the head. I ventured to give sal volatile, brandy, and nourishment. We had, in a short time, the pleasure of observing the eye-lids become impressible to the stimulus of the finger, the respiration to become regular, and the gradual recovery of the little patient was no longer doubtful." 93.

*Mania with and without Paralysis.*—Dr. Hall alludes to M. Leuret's position, that mania without paralysis is altogether unattended with organic lesion. He believes that there is a lesion of an *intra-vascular* kind. We fancy this opinion is not confined to Dr. Hall.

*Tetanus.*—This disease, says our author, is, in every respect, the most unequivocal example of an affection of the true-spinal marrow, through an incident and the motor nerves. All the functions of this sub-division of the nervous system are affected in the most violent form, whilst the cerebral functions are unaffected: the dyspnœa, the dysphagia, the constipation, the trismus, the emprosthotonos, the opisthotonos, the extreme susceptibility to causes of physical impression and agitation, and of mental emotion,—all mark an affection of the true-spinal system; whilst the freedom from all affections of the senses and of the intellect, the absence at once of delirium and of coma, denote the normal condition of the cerebral system. Hydrophobia is in the same category.

*Epilepsy.*—Dr. Hall thinks that in this the very first symptom is generally, if not always, one of the true-spinal kind. This first symptom is constriction about the throat, and closure of the larynx, more or less complete; then follow violent expiratory efforts and convulsive movements of the trunk and limbs. Intermediately, and even without the convulsive movements, the cerebrum is affected with congestion, and a multitude of cerebral symptoms occur: flashes of light, tinnitus aurium, the aura epileptica; a momentary oblivion; a state of terror, of delirium, or of unconsciousness, &c.; as *parts* of the general convulsion, the tongue is protruded and bitten, the fæces, the urine, or the semen expelled; as *consequences* of that convulsion, the cerebrum is congested, and there is coma. If this state continues, another order of symptoms takes place; the respiration becomes stertorous, and, at length, affected with mucous rattle, the true-spinal and ganglionic systems becoming fatally involved in the disease.

It is the constriction about the throat which assimilates epilepsy to the state of things which exists in *strangulation,* and which distinguishes it from hysteria. It is this circumstance which associates epilepsy with the crowing inspiration and the convulsions of children; all are *laryngismal.* In epilepsy, there is sometimes a crowing inspiration; the crowing inspiration and convulsion of infants are sometimes followed by epilepsy in subsequent years.

*Sinking.*—The gradual sinking from loss of blood, especially, seems, says Dr. Hall, to involve every part of the nervous system. There is mild delirium or stupor from affection of the cerebral system; there is a peculiar catching motion of the larynx and other organs of respiration, from affection of the true-spinal system, instead of the *equable* rhythmic movements observed in health; there is an extreme frequency of the pulse; and there is a peculiar crepitant rattle, at first in the small, and eventually in the larger bronchi, and an equally peculiar tympanitic distention of the intestines, from affection of the ganglionic system, *all* of fatal import. The functions of the cerebrum, of the true-spinal system, and of the ganglionic system, seem to fail altogether.

*Shock.*—The effects of *shock* have their chief seat in the ganglionic system—the circulation, the secretions are greatly affected.

"In the presence of a young Parisian student, I divided the spinal marrow in a frog. I pinched the toes, but there was no movement, no reflex action. My companion observed, 'Ah, c'est fini;' I replied, 'Non, ce n'est pas commencé.' In a few minutes, the reflex actions became obvious, and in a few minutes more, most energetic. We had examined the circulation previous to the division of the spinal marrow. It was most active. But immediately after that division, scarcely a movement was to be seen. Like the reflex actions, however, the vigour of the circulation was gradually restored.

In one frog, after the return of the circulation, I crushed the leg and thigh with a hammer. There was no sensation of course, the influence of the cerebrum having been removed. The circulation again immediately ceased. It again returned after a time." 99.

2. *The Influence of Irritation, of Pressure, of Counter-irritation, and of Counter-pressure, in Disease within the Cranium and Spinal Canal.*

After remarking on the important part played by irritation and pressure in diseases of the nervous system, Dr. Hall adds:—" Not less important, and hitherto overlooked or neglected, are *counter*-irritation and *counter*-pressure, of which I shall therefore proceed to treat more particularly. The former is induced by slighter causes, as slight effusion into the ventricles, the latter, by the same causes carried to a greater degree."

*Counter-irritation.*—Dr. Hall thus attempts to explain a fact stated by Andral, and familiar to all, that the same cerebral lesion in different cases will produce different symptoms. He says:—

" M. Andral speaks of *irritation* of the *cerebrum* as the cause of abnormal muscular contractions. Now, in our investigations into the nature of cerebral diseases, we must remember one circumstance; it is impossible to induce muscular action by any irritation of the substance of the cerebrum itself. Whenever, therefore, there are spasmodic affections in diseases of the nervous system, we *must* conclude that the *spinal* system is involved, either primarily or secondarily, in the disease. Irritation of the cerebrum may induce delirium and other disorders of the cerebral functions; congestion of the cerebrum may induce coma, paralysis, &c. But if these morbid conditions of the brain be attended by spasmodic or other deranged actions, it is because the true-spinal system is involved in the disease, or affected by it in the way of irritation, counter-irritation, or of pressure, or counter-pressure. Hence we observe the symptoms of spasm in various diseases of the encephalon, the condition being, not the nature of the disease, but that they produce these intermediate effects. *Time,* as is well known, is a very important element in this problem; and why is it so? The fact is to be explained on the same principles. The very same lesion occurring quickly, will produce effects which will be totally absent if it creep on slowly. In the former case, we have the effects of irritation and pressure, or of counter-irritation and counter-pressure; in the latter, the cerebrum has so accommodated itself to the new state of things, probably by the altered condition of its vessels, as to avoid these effects, except towards the close of the disease.

We need not, therefore, now view with surprise the fact that the same lesion, as found *post-mortem,* had been attended by a totally different series of symptoms during life, any more than the other fact, that, in the different *periods* of that lesion, the symptoms have been different.

The symptoms frequently subside too and re-appear. If the disease be not regularly progressive, the encephalon accommodates itself, as I have stated, and the symptoms disappear; if now the disease proceeds, the symptoms also return. At least all this *may* be.

A rapid effusion of serum may resemble hæmorrhage or ramollissement in its effects; or slow effusion may merely obscure the intellectual faculties." 105.

It is not, therefore, says Dr. Hall, the *disease,* but its *effects* upon the brain and spinal marrow, which is the source of the symptoms. If ramollissement, effusion, a tumor, &c. produce similar effects on these textures, the same affection of the functions, the same symptoms, will be observed.

However ingenious this explanation, it leaves the *practical* difficulty where it found it. The difficulty in question is this:—given, any set of symptoms, what is the lesion? Such symptoms are found in conjunction with such different lesions, or independent of any which can be appreciated, that precision of diagnosis seems impossible.

*Counter-pressure.*—Dr. Hall comments on the effects of undue pressure, and of defective pressure.

" It is well known from the experiments of M. Flourens especially, that irritation of the cerebrum has no influence in inducing spasmodic action. Whenever, therefore, spasmodic symptoms occur in diseases of the cerebrum, it must as I have already stated, be on a principle different from that of irritation of the substance of the cerebrum itself; it must be from an impression made upon parts of the nervous system in which the property of exciting spasmodic action on being subjected to irritation resides; these parts are the tubercula quadrigemina, the medulla oblongata, the intra-cranial nerves, &c.

That *undue* counter-pressure on the medulla oblongata may, and actually does, excite convulsion, is proved by the following facts: In the interesting case, most anxiously watched and accurately detailed to me by my friend Mr. Toogood, of Bridgewater, of his own little girl, aged thirteen months, the croup-like convulsion occurred repeatedly, until one day, when the bones of the cranium separated, and the convulsion ceased. In a case of spina-bifida, related to me by Mr. Herbert Evans, of Hampstead, there was a croup-like convulsion whenever the little patient, turned so as to press upon the tumor. In the case of an anencephalous fœtus, described by Mr. Lawrence, convulsion was produced on pressing on the medulla oblongata. In a case of meningitis, given by Dr. Abercrombie, the anterior fontanelle became very prominent. Pressure upon it induced convulsion. Hypertrophy of the brain affords an argument of the same kind: it induces convulsion, *except* in the case in which the cranium grows with the encephalon. These and other facts lead me to think that convulsion arising from cerebral disease is thus to be explained." 107.

Dr. Hall relates an interesting case from Andral, in which defective pressure was a cause of convulsions likewise. M. Berard removed a fungous tumor from the dura mater—convulsions followed. He applied pressure on the cerebrum, and the convulsions ceased.

Dr. Hall concludes, that the true spinal symptoms which occur in cerebral attacks arise from counter-pressure; when the source of this is permanent, as in hæmorrhagy, the effect is permanent too, and the case fatal; when it is remediable by bloodletting, as in congestion, the cause and its effects are removed together.

The convulsions induced by hæmorrhage depend upon a similar subtraction of the intra-vascular pressure of the blood in the medulla oblongata. But the changes must be rapid. The cerebrum accommodates itself to tardy ones.

The next subject to which Dr. Hall passes is:—

### 3. *The Special Anatomy of the Base of the Encephalon in reference to Diseases of the Nervous System.*

After noticing the disposition of the constituents of the basis of the brain, and the effects of the tentorium in preventing direct pressure from the cerebrum on the parts below; Dr. Hall adds:—

" It is these circumstances, combined with another element of the proposition—that of *time*,—which frequently leads to an effect which I shall notice immediately; viz. the difference of symptoms with identity of lesion, and the similarity in the symptoms when the lesion is dissimilar. The same morbid change will produce very different effects, developed as an acute and as a chronic disease; and different physical lesions will produce nearly the same results if developed in nearly *equal times*.

In a chronic affection, the cerebral substance yields, its vessels becoming empty, and pressure is not induced. In acute affections, on the contrary, pressure is made upon contiguous, and counter-pressure upon distant parts, with their appropriate symptoms. By degrees, even in the latter case, the cerebral substance yields, and the symptoms, in the less severe cases, subside, and even disappear." 110.

Then comes an important inquiry, viz,—

4. *Why, with similar symptoms, have we dissimilar morbid appearances, and vice versâ?* and, *what are the Diseases of the Nervous System, in which we find no morbid appearances on a post-mortem examination?*

If, observes our author, the source of the symptoms be not the mere lesion of a function, induced by the lesion of a special part or organ of the encephalon, but the effect of irritation and counter-irritation, of pressure and counter-pressure, it is obvious that these primary effects, and their effects in their turn, may result from *any* disease, if the *times* be similar, whatever that may be.

It is accordingly to the *history* that we chiefly have recourse for the diagnosis of cerebral diseases, and especially to that of the seizure and first stage: at their close, almost all diseases of the encephalon are alike: almost all terminate by coma, paralysis, convulsions, stertor, and impaired actions of ingestion and egestion, and of the orifices and sphincters, from compression of the cerebrum and medulla oblongata.

If Dr. Hall implies that different lesions occurring in the same *time have* the same symptoms, we fear we must hesitate to agree with him. If he contends only that they *may* have, that is another matter. One case of chronic abscess in the cerebrum may be attended with obstinate vomiting—another case of abscess, equally chronic, may never be accompanied by vomiting at all. No doubt, the discrepancies depend on modifications of irritation, pressure, vascularity, &c. but the capability of measuring or determining in diagnosis those seemingly capricious and fugitive conditions, will probably ever be denied to us.

Besides, as Dr. Hall remarks, morbid changes take place towards the *close* of many diseases, which do not properly, or at all constitute the disease. In exhaustion, in chlorosis, in delirium tremens, effusion of serum, and even of lymph, occurs. In disease of the encephalon itself, such effusion also takes place, late in its course, and *complicates* the original disease.

*Effects of Exposure to Severe Cold.*—Whilst exposure to a moderate degree of cold conduces to the state of hibernation, a physiological and preservative condition, exposure to intense cold induces *torpor*, a state totally different, but not sufficiently distinguished from the former, of a pathological character, and of *fatal* tendency. In the state of hibernation, the animal is dormant and motionless, but the actions excited are perfectly regular; in the state of torpor, on the contrary, the animal moves about, but the movements are, in the highest degree, irregular and tottering. This stupor ends in death.

In man, the impairment of muscular power, when benumbed by cold, is well known.

Exposure to extreme heat or cold equally induces spasmodic action in the muscular system. A young gentleman having been ordered a warm bath, mistook the temperature, and exposed himself to such a degree of heat as induced general spasmodic action of the most painful kind. The effect of too intense a cold on swimmers is familiar.

"When the exposure to cold is more partial, effects on both the sentient and motor portions of the nervous system are produced, which have this characteristic:—there is at first paralysis, and afterwards undue action. The first effect of exposure to cold is numbness in the fingers; this usually yields to pain, vulgarly termed 'hot-ache,' especially if the warmth be restored too rapidly. In a relative of mine, exposure to a severe wind, with sleet, induced perfect numbness of one side the face; this paralysis subsided, and gave way to severe tic douloureux. A lady, whose case I shall detail more at length immediately, was exposed to severe cold with wind. The next day she arose from bed with paralysis of one side of the face! This paralysis yielded by degrees to spasmodic tic.

Exposure to cold is a far more frequent cause of paralysis than is generally supposed. Such an effect on the face has been designated, in common language, (which frequently involves an important truth,) *a blight*. Cases of paralysis of the face, from exposure to cold, are detailed by Dr. Powell in the fourth volume of the Transactions of the Royal College of Physicians. There is a poor little boy, residing near me, of six years of age, whose limbs are nearly paralytic in consequence of a long and most criminal exposure to cold by a nurse. Some years ago, I visited a gentleman perfectly paraplegic, from long exposure to intense cold on the outside of a coach. Baron Larrey speaks of permanent paralysis, left by exposure to intense cold during the campaign in Russia. Paralysis, happily of a less permanent character, has been experienced by every one under similar circumstances.

But the point to which I must now revert, and to which I beg to call the attention of the members of this Society, is, that the first effect of a partial but severe exposure to cold is paralysis; whilst the more remote effect is undue action." 118.

Dr. Hall relates a case in which the face was first drawn to the *left* side, the *right* eyelid being paralysed. Afterwards the face was drawn to the *right* side, the *right* eyelid being closed spasmodically. He concludes, and probably with truth, that there was first a paralytic condition of the *right* facial nerve, and afterwards a spasmodic affection of it. From misapprehension of this, the remedies were applied to the wrong side of the face.

In conclusion, Dr. Hall recommends that, in all future cases of disease of the nervous system, the various points be observed in the following order.

I. *The Cerebral Symptoms.*
  1. *Excess, or defect, in the Senses; Pain.*
  2. *Delirium; Coma.*
  3. *Paralysis.*

II. *The true-Spinal Symptoms.*
  1. *Spasm, clonic or tonic.*
  2. *Paralysis,—in regard to*
    1. *The functions of Ingestion.*
    2. *The functions of Excretion.*
    3. *The Muscular System generally.*
  3. *Reflex and Retrograde Actions.*
  4. *Irritability of the Muscular Fibre.*

III. *The Ganglionic—in regard to*
  1. *Nutrition.*
  2. *Temperature.*
  3. *The Secretions, especially those of*
    1. *The Bronchi.*
    2. *The Stomach and Intestines.*
    3. *The Kidneys and Bladder.*

IV. *The Effects of Emotion.*

V. *The Effects of Shock.*

VI. *The Effects of Counter-pressure, &c.*

Like all Dr. Hall's Memoirs highly ingenious.

### IX.—On Dislocations, especially of the Hip-Joint, accompanied by Elongation of the Capsule and Ligaments. By *Edward Stanley*, F.R.S.

Mr. Stanley's object is to direct attention to the subject of dislocations of the larger joints, and especially of the hip, occurring under other circumstances than as the direct consequence of external violence, or of the destructive processes of inflammation. Mr. Stanley relates seven cases. We will give them as briefly as we can.

Case 1.—*Dislocation of both hip-joints, consequent on disease of the spinal cord, and probably of the brain.*—A gentleman, aged thirty-nine, in the year 1824, was attacked with spasms in the pectoral and intercostal muscles, and numbness in the whole of the left side of the body, with the exception of the arm. In the left thigh and leg, sensation was wholly lost, the power of motion remaining. He had no sensation of passing his urine after it had quitted the bladder, and was but just aware of the evacuation of the fæces. Vision in the left eye was impaired to the extent that he could but distinguish daylight. These symptoms continued, with increasing weakness in the thighs and legs, to the complete loss of the power of support, and of sensation in them. With the impairment of the natural sensibility of the limbs, he occasionally suffered in them the most severe pains, sometimes attended with a smarting sensation, at others, with the sensation of a blow frequently repeated.

In March 1828, he was attacked with violent spasms in his body and limbs, which compelled him to remain in bed several days. On rising from his bed when the spasms had subsided, he found, to his great surprise, his right lower limb so much shortened, that when erect he was but just able to touch the ground with his great toe, and at the same time he remarked a protuberance at the upper and back part of the thigh. In the following December there was a repetition of these occurrences, but in the other limb, an attack of spasms being followed by shortening of it, with a protuberance at the back of the thigh, as on the opposite side. He could still bear the weight of his body upon his limbs, but was almost wholly unable to move them. At no period had there been tenderness, or other sign of inflammation in the soft parts around the hip-joints.

In June 1831, Mr. Stanley noted the following particulars:—The spasms and the attacks of pain are chiefly confined to the chest and to the lower limbs; he suffers a distressing sensation of tightness with acute pain on both sides of the chest, in the direction of the ribs from their angles to the sternum. Movements of the arms excite this pain. Firm pressure by the hands against the walls of the chest greatly relieves it. There is paralysis of the rectus superior muscle of the left eye, and its sensibility to the impression of light is much weakened. In the erect posture there is a remarkable projection at the back of the pelvis, which, upon examination, is ascertained to be caused by the extremities of the thigh-bones occupying this situation. In rotating each thigh, the head of the femur can be felt moving freely beneath the glutei muscles. The trochanter major of each femur is thrown directly backwards. The distance between each trochanter and the head of the bone is natural. The head of each femur thus situated upon the posterior part of the pelvis is two inches and a half below the highest part of the crista of the ilium, and four inches distant from the anterior superior spine of the same bone. In the erect posture, there is a diminution in the stature to the extent of between five and six inches, and evidently from the pelvis sinking between the thighs. In the horizontal posture, the thighs can be readily pulled downward so nearly to their natural situation, that the shortening of the stature is then only to the extent of between one and two inches.

At the present time, the extremities of the thigh-bones are not so moveable as they were, apparently from the thickening and consolidation of the surrounding cellular tissue. There has been a gradual recovery of the power of directing the movements of the limbs, which is now sufficient to enable him to walk at a slow pace, with the aid of a stick.

Case 2.—*Dislocation of the hip-joint consequent on an attack of hemiplegia.*—A gentleman aged 48, had been for above eight years affected with hemiplegia, chiefly perceptible in the left lower limb. He had been a courier, and he attributed his complaint to the severity and vicissitudes of weather to which he had been constantly exposed. Two years before his death, it became evident, as he moved about on crutches, that the affected limb had become considerably lengthened; this was accompanied by wasting of the limb, with a remarkable flaccidity of the muscles; and on rotating the thigh, the head of the femur could be so plainly felt, that it was concluded it must be out of its socket. This circumstance gave an interest to the case, which led to a careful examination of the hip-joint after death, when the following peculiarities were noticed: The capsule and the ligamentum teres were entire, but elongated to the extent of allowing the head of the femur to pass beyond the limits of the acetabulum.

CASE 3.—*Dislocation of the hip-joint consequent on rheumatism.*—Mary Elsely, aged 32, admitted into St. Bartholomew's Hospital, December, 1837, on account of general febrile disorder, combined with pain in the left shoulder-joint, in the right hip-joint and down the front of the thigh. She was in the fifth month of pregnancy; her occupation was that of a hawker of brushes, which exposed her to the vicissitudes of the weather. She stated that she had been in good health until within a fortnight of her admission, when she was attacked first with pain in the right elbow and left shoulder-joints, and afterwards in the back of the right thigh. The pain varying in severity, was occasionally acute; on some days it was confined to the thigh, and on others was excruciating through the whole limb from the hip to the toes. Throughout, there had not been more constitutional disturbance than might be referred to the amount of pain she endured. Her disorder was treated as rheumatism. Her complaints slowly subsided, and more than ten weeks had elapsed before she was able to quit her bed. On doing so, she discovered a shortening and distortion of the right lower limb. There was a dislocation of the head of the femur on the dorsum of the ilium.

CASE 4.—*Dislocation of the hip-joint consequent on pain in the thigh, treated as sciatica.*—A woman, aged 30, was admitted into St. Bartholomew's Hospital in March, 1838. She had been servant in a gentleman's family at Hampstead, where her illness had commenced with a painful swelling of one of the joints of the right thumb, which, after a day or two, subsided, and was immediately succeeded by pain and stiffness in the right hip-joint, so far impeding its motions, that she was occasionally confined to her bed. She was seen at this period by several medical men, who considered the case to be sciatica, and treated it as such for between two and three months before her admission into the hospital. She now complained of pain in the right hip, but extending upwards to the loins, and down the thigh, with stiffness of the whole limb. The skin was tender on pressure through the course of the ischiatic nerve. Succussion of the whole limb by the application of force to the sole of the foot, and with the knee-joint extended, produced no pain in the hip-joint; and although the movements of the hip were impeded by the pain in the thigh and the general stiffness of the limb, yet she could bear the weight of her body upon it without much inconvenience. In the view still taken of the disease, as being sciatica, no other treatment was adopted than the administration of opium, with the application of mustard poultices to the limb. This treatment having been continued for some time without benefit, she was removed to a surgical ward, when there was discovered to be a complete dislocation of the affected hip. This must have occurred in the hospital. There was no inflammation, but nervous excitement which gradually subsided. An attempt, of dubious utility, was made to extend the limb gradually by a pulley and weight.

CASE 5.—*Dislocation of the hip-joint consequent on rheumatism.*—"I was consulted in June, 1836, respecting the propriety of attempting to reduce a dislocation of the hip-joint which had occurred under the following circumstances. A youth, aged fourteen, suffered, at Gibraltar, an attack of inflammation in both hip-joints, with severe constitutional derangement, which was reported to be of the nature of rheumatic fever. He was confined to his bed above twelve months. Then, on beginning to move about, it was discovered that the right hip-joint had become dislocated, but he was wholly unaware when the dislocation had occurred. At the time of my seeing this patient, four years had elapsed since the commencement of his illness. His general health is now perfectly good. There is not the slightest pain in the hip or elsewhere, but the limb is everted and shortened, as ascertained by exact measurement, to the extent of three inches and a half. The trochanter major is greatly more prominent than in the opposite limb, and the head of the femur is readily distinguished through the glutæi muscles, having its proper relation to the trochanter consistent with the integrity of the neck of the bone, and with the everted position of the limb. Flexion and extension of the thigh are perfectly free. Rotation of the thigh inwards can be executed, but not outwards. The soft parts of the hip, and in its neighbourhood, are sound." 133.

Mr. Stanley did not attempt reduction.

Case 6.—*Dislocation of the hip-joint, which occurred in the sixth week from a fall.*—A female, aged 14, in passing through a passage, the stones of which were slippery, fell upon the outer side of the right thigh. There immediately ensued a powerless condition of the limb, which was soon followed by severe pain and swelling in the front and outer part of the thigh, with spasms of the muscles. The surgeons summoned to the case could detect no deviation from the proper length and position of the limb, and accordingly expressed their opinion that the injury was confined to the muscles. At the expiration of a month there was no recovery of the power of using the limb, and the patient was in consequence removed to the sea side. Gentle efforts to walk were here made with the help of a stick, and at the same time the limb was every day placed in a vapour bath. At this period the patient occasionally remarked that she thought her hip was growing out; on one occasion, whilst using the vapour bath, she observed to her attendants that the projection of the hip had suddenly increased, and on examining the limb immediately afterwards, there was found to be a well-marked dislocation of the head of the femur. How this dislocation had occurred no opinion could be given; but the surgeon who had been in daily attendance was certain no dislocation had existed before the present time, which was in the sixth week from the occurrence of the fall. There was no tendency to inversion or eversion of the limb, and it could be moved freely in any direction, when the head of the bone might be felt rolling beneath the fingers placed upon the hip. The neck of the femur could be distinguished, and of its integrity there could be no doubt, from the movement of the head of the bone simultaneously with the trochanter, and from the preservation of the natural distance between the two prominences. Nothing but quietude was recommended. When about six months had elapsed from the occurrence of the accident, the patient, on rising from her bed, exclaimed that the projection of the hip had disappeared, and that her limbs were of the same length. A careful examination of the injured limb confirmed the statement of the return of the head of the bone to its socket, but it subsequently became again displaced; for at a later period, the head of the femur could be plainly felt on the dorsum of the ilium, and the limb was now shortened to the extent of three inches, but still neither inverted nor everted. The power of using the limb was however progressively increasing.

CASE 7.—*Injury to the hip-joint, attended with shortening of the limb, from a fall upon the knee.*—A youth, aged 18, in walking, was thrown down by his foot striking against a pole which lay unperceived in his way. His face and left knee were the only parts bruised. But on being raised from the ground, he was unable to bear weight upon the left leg, and felt pain in the upper part of the thigh. He remained in bed until the pain in the thigh ceased, and then, on moving about, the limb was, as he stated, very feeble. Three months after the accident, the limb was in this state. By comparison with the sound limb, there was found to be a diminution of the space between the anterior superior spine of the ilium and the top of the patella to the extent of two inches. There was no inversion or eversion of the foot. The head of the femur could not be anywhere distinctly recognized. The trochanter major was considerably more prominent than on the opposite side. All the movements of the thigh could be freely executed, and without pain. By moderate extension with the hands, the limb could be brought down to its natural position, when the unnatural prominence of the trochanter disappeared; but on remitting the extension, this prominence reappeared, and the limb became again shortened. No thickening or other morbid change could be discovered in the soft parts around the hip-joint.

Mr. Stanley remarks on the preceding cases :—

"In the first and second cases which have been related, the displacement of the head of the femur from the acetabulum occcurred as a consequence of impaired nervous power, combined with spasms in the muscles of the limbs, in one case ascertained to be from disease of the spinal cord; and in the other, presumed, from the collateral symptoms, to be from the same cause. It may be affirmed that in the first, and very remarkable case, where both hip-joints were dislocated, there had been, at no period, inflammation in the joints or contiguous parts; and under such circumstances we must, I think, conclude that the pathological changes in these joints had been the elongation of their capsules and ligaments. In the second case, dissection showed such to be the condition of the joint, the capsule and ligamentum teres being lengthened to the extent of allowing the head of the femur to pass considerably beyond the acetabulum. We know that lengthening of the arm may be the consequence of paralysis of the deltoid and other muscles combining in their natural actions, to maintain the articular surfaces in contact. It may be said that the looseness and thinness of the capsule of the shoulder-joint permit no comparison of it with the dense, thick, and closely-embracing capsule of the hip-joint. However this may be, we have before us the fact of the lengthening of this capsule, and with it of the ligamentum teres, of which no other account can be rendered than that it was a consequence of impaired nervous power in the muscles surrounding the articulation.

In the third, and in the fifth case, the dislocation of the hip must be viewed as the consequence of rheumatic inflammation in the fibrous and synovial tissues of the joint; and in the fourth case, the dislocation may be ascribed to the same cause, although the disease had been treated under the name of sciatica. It can scarcely be a question, that in each of these three cases, the pathological changes were elongation of the capsule, with either the elongation or actual destruction of the ligamentum teres. The sixth and seventh cases are examples of injuries to the hip-joint from external violence in young persons, followed by a gradual shortening of the limb, which, from the attendant circumstances, can be explained only by the yielding and consequent lengthening of the ligamentous tissues of the joint. In the sixth case, the head of the femur was gradually, and at a distant period from the injury, displaced from the acetabulum. In the seventh case, a similar change in the relations of the articular surfaces was indicated by the shortening of the limb, although the head of the femur could no where be distinctly recognized. Other cases have been reported to me of dislocations of the hip-joint, occurring gradually, and without inflammation, after injuries from external violence. Whatever difficulty there may be in explaining such a form of dislocation, the knowledge of the simple fact of the possibility of its occurrence is of much importance to the establishment of a correct diagnosis of the various injuries occurring to the hip-joint." 140.

Mr. Stanley goes on to remark, that it is well ascertained that inflammation of a mild character, whether rheumatic or otherwise, may, without evident change in the organization of ligamentous tissue, so far affect its property of resistance, that it will yield considerably to an extending force; thus, in the knee-joint, the crucial and lateral ligaments may become lengthened to the extent of permitting such a displacement of the articular surfaces, that, from the view of the outside of the joint, it might be inferred actual destruction of the ligaments had taken place; and it is to be observed, that these changes in the ligaments of a joint, very slow in progress, may be unaccompanied by pain or other symptoms of inflammation.

In the hip-joint, again, from inflammation of a mild character, and probably commencing in its fibrous tissues, there may be effusion of fluid into the capsule with the yielding of it, and of the ligamentum teres producing, first, an increased length of the limb, and an increase of its circumference in the district of the joint; and subsequently, on the head of the bone reaching the brim of the acetabulum, a shortening of the limb, as the capsule gradually yields to the action of the powerful muscles constantly tending to draw the limb upwards and backwards.

Mr. Stanley mentions a remarkable instance of *voluntary* dislocations of the hip, in a person whose capsules were preternaturally elongated by exercise. A boy, aged 18, was sent to St. Bartholomew's Hospital, in whom the following particulars were observed. His muscular system was remarkably well developed. When standing erect, he could, by the action of the muscles, throw the head of either femur out of its socket to the back of the pelvis, where it was felt projecting as in the ordinary dislocation from external violence, and as readily, still standing erect, he could, by renewed muscular effort, throw the head of each bone back again into its socket. It was remarkable that with such a degree of motion in the hip-joints, neither the firmness of his erect position, nor his power of progression, was in any degree impaired. They learned that he had been exhibiting feats at a country fair.

Mr. Stanley adverts to the supposition that in a proportion of the cases described, the primary injury was rupture of the ligamentum teres. This is rendered improbable by occasional absence as well as rupture of that ligament without injurious consequences.

Mr. Stanley adds, with a lengthening of the capsule of the hip-joint, it is unlikely that the head of the femur would be displaced in any other direction than upwards and backwards, with a corresponding shortening of the limb, the action of the more numerous and powerful muscles tending to this result; and it may be presumed, that the precise situation of the head of the bone will then be between the glutæus minimus muscle and the dorsum of the ilium, An exception to this would occur in the yielding of the capsule consequent on a paralytic condition of the muscles, when an increased length of the limb may be its permanent character, as in the second case which has been related. With the lengthening of the capsule and the passage of the head of the femur upwards and backwards to the dorsum of the ilium, there may be inversion or eversion of the limb, or no inclination of it to one or other position. Whether these differences depend on the condition of the ligamentum teres, as this may be elongated or removed, future observation must determine. It will be remarked, that in the majority of the cases which have been related, the displacement of the head of the femur occurred so gradually, and with such a freedom from uneasiness in the part, that the patient was wholly unaware that changes so important were in progress; in fact, there was no suspicion of them before the discovery that the dislocation had actually taken place. The remarkable mobility of the limb in most of these cases is also to be noticed as another distinctive character of these displacements when contrasted with the ordinary dislocations of the hip-joint consequent on external violence, or on disease.

The preceding, like all Mr. Stanley's Papers, is indicative of his sound observation and sense.

### X.—OBSERVATIONS ON THE ANATOMY OF THE LUNGS. By *Thomas Addison*, M.D.

Dr. Addison, than whom no man better merits attention, tells us that he hopes he has succeeded in demonstrating, almost beyond dispute—1st. that the aerial cellular tissue of the lungs is made up of well-defined, rounded, or oval lobules, united to each other by interlobular cellular membrane, each lobule constituting a sort of distinct lung in miniature, having its own separate artery and vein; 2ndly, that these lobules do not communicate directly with each other; 3rdly, that they do not, as Reissessen and others have supposed, consist of the globular extremities of as many bronchial tubes, but, on the contrary, as my friend Dr. Hodgkin has suggested, are made up of a collection of cells, in which, by a common opening, a minute filiform bronchial tube abruptly terminates; 4thly, that the pulmonary artery accompanies the bronchi, branch for branch, to the minutest divisions of the latter; 5thly, that pneumonia consists essentially in inflammation of the aeriel cells; 6thly, that pneumonia and inflammatory tubercle are identical; 7thly, that acute pneumonia in moderately good constitutions scarcely ever leads to the formation of an abscess, unless deposit previously existed; but that when it occurs in cachectic or broken-down constitutions, or supervenes in the process of chronic or organic diseases, it occasionally causes one or more distinct and separate lobules to soften down into an ill-conditioned abscess; 8thly, that ordinary tubercles present the same varieties in the lungs, as they do in serous membranes; 9thly, that emphysema of the lungs consists chiefly of mere dilatation of the cells, but in part also sometimes of more or less extensive laceration of them ; and lastly, that the circumscribed gangrene of Laennec is commonly, if not uniformly, a mere effect or advanced stage of pulmonary apoplexy.

His present object, however, is merely to point out a hitherto unnoticed mode of distribution of the pulmonary vein.

In order, says Dr Addison, to accomplish this demonstration, the pulmonary artery was injected with size, coloured red, whilst the vein was injected with the same material, coloured yellow : the lung was then laid aside and kept moistened in a cool place for several days, with the view of softening, by approaching decomposition, the connecting cellular membrane distributed throughout the lungs. In this way the common cellular membrane beneath the pleura became so lacerable that the pleura itself was stripped off without much difficulty, and without inflicting any breach whatever in the aeriel cellular structure of the lung, which it had covered. The lung thus divested of its pleura, presents to the eye, more or less distinctly, lines on its surface, which indicate the situation of what may be called the *pulmonary fissures*—a term more correctly applicable than that of interlobular, inasmuch as by the term interlobular is usually understood a something situated between either the longer lobes or smaller lobules; whereas by the term *pulmonary fissures*, is meant certain spaces, occupied by common cellular membrane, and which descend from the surface towards the interior, but without penetrating the aeriel cellular tissue of the lung; thereby dividing more or less deeply the surface of the organ into a number of insular portions, some of which may comprise a great number of lobules. Guided by the linear indications on the surface of the now naked lung, we can in general, with the aid of a pair of points let into handles, or a pair of fine scissors, and without much difficulty, succeed in laying open and exposing the pulmonary fissures, at the bottom of which, merely surrounded by a loose cellular membrane, and resting on the unbroken aeriel pulmonary tissue, we discover a vessel ; that vessel is the pulmonary vein, alone, and unaccompanied by any artery whatever. This vessel may be distinctly traced from larger to smaller trunks towards its source, until we reach the common cellular membrane between the ultimate lobules, from the exterior of which the vein appears to originate ; whilst on the other hand, by continuing the mechanical operation towards the root of the lungs, we, with almost equal facility, trace the vessel, still lying at the bottom of the pulmonary fissures, and becoming gradually larger and larger by the addition of branches, which proceed into the pulmonary fissure, and are derived either from the neighbouring smaller pulmonary fissures, or from the uniting cellular membrane between the ultimate lobules themselves, until at length it joins the large trunks at the root of the lungs, to form the great pulmonary veins. A small artery is not unfrequently observed running across the pulmonary fissures, from a portion of lung on one side to a portion of lung on the other; and in one instance, Dr. A found an exceedingly narrow strip of healthy lung passing like a bridge across the fissure, on the very surface of the lung.

Thus, then, the human lung may be said to be made up essentially of a vast expanse of membrane, the interior of which, during the whole of extra-uterine life, is unceasingly exposed to the influence of atmospheric air, and upon the surface or in the substance of which, are spread out the capillary ramifications of the pulmonary artery; these arterial capillaries passing from thence to the exterior of the membrane, to form the pulmonary vein, which throughout its whole course is found to be situated on the exterior of the aerial cellular structure of the organs.

### XI.—RESULTS OF AMPUTATIONS AT UNIVERSITY-COLLEGE HOSPITAL, LONDON, STATISTICALLY ARRANGED. By *John Phillips Potter*, Esq., late House-Surgeon. WITH SOME REMARKS ON THE MODE OF AMPUTATION AND METHOD OF DRESSING THERE ADOPTED. By *Robert Liston*, Esq.

The Reporter very properly insists on the utility of Statistical Reports from Hospitals and urges their more extensive publication. In this we cordially agree with him.

The number of cases of amputation in the University-College Hospital, from the last day of June 1835, to the termination of the year 1840, a period of six years and a half, has been 66, and of these, 56 have proved successful, whilst 10 have been attended with fatal results, at a variable period of time after the performance of the operation.

Of the 66 cases, 11 were subjected to amputation on account of severe compound fractures, and other injuries, the operations having been performed within 24 hours after the occurrence of the accident.

Out of these 11 cases of primary amputation, 3 terminated fatally, one in 7 days, one in 11 days, and one after the lapse of 48 days. The first of these fatal cases was of an unusually severe character, the patient having received, on the Birmingham Railway, compound fracture of both legs, fracture of the humerus, fracture of the ribs, and several severe contusions. In this instance both legs were removed, one in a few hours after the receipt of the injury, and the other a few days afterwards, in consequence of traumatic gangrene.

Seven out of the 11 cases recovered, and the stumps were healed completely, after the following times:—in 23 days, 30 days, 35 days, 48 days, 61 days, 75 days, and 1 after 146 days.

Table I, gives the parts which were amputated, and the results in each; from which it would appear that amputations on the lower extremity are, as might be expected, more dangerous to life than those of the upper extremity.

In the remaining 56 cases, amputation was performed on account of long-standing disease, or for injuries in which an attempt was made to save the limbs. Of this number, only seven died, giving a proportion of one death out of 8 cases.

Tables II. and III. give the parts at which amputation was performed, the nature of the different cases, and their results, together with the proportion of deaths under these different circumstances.

TABLE I.

| | Cases. | Cured. | Died. |
|---|---|---|---|
| Arm .. .. .. .. .. .. .. .. | 1 | 1 | 0 |
| Fore-arm .. .. .. .. .. .. .. | 2 | 2 | 0 |
| Wrist.. .. .. .. .. .. .. .. | 1 | 1 | 0 |
| Thigh.. .. .. .. .. .. .. .. | 2 | 1 | 1 |
| Leg .. .. .. .. .. .. .. .. | 3 | 2 | 1 |
| Both Legs.. .. .. .. .. .. .. | 1 | 0 | 1 |
| Total .. .. | 10 | 7 | 3 |

*Proportion of Deaths barely* 1 *in* 3 *cases.*

TABLE II.—56 *Cases of Secondary Amputation.*

| | Number of Cases. | Cured. | Died. | Prop. of Deaths. |
|---|---|---|---|---|
| Shoulder .. .. .. .. .. .. | 1 | 1 | 0 | |
| Arm .. .. .. .. .. .. .. | 7 | 5 | 2 | 1 in $3\frac{1}{2}$ |
| Fore-arm .. .. .. .. .. .. | 6 | 6 | 0 | |
| Thigh.. .. .. .. .. .. .. | 20 | 17 | 3 | 1 in $6\frac{2}{3}$ |
| Leg .. .. .. .. .. .. .. | 22 | 20 | 2 | 1 in 11 |
| Total.. .. | 56 | 49 | 7 | |

TABLE III.—*Nature of the* 56 *Cases of Secondary Amputation.*

| Nature of Case. | No. of Cases. | Cured. | Died. | Prop. of Deaths. |
|---|---|---|---|---|
| Diseased joints . . . . . . . | 33 | 28 | 5 | 1 in $6\frac{3}{5}$ |
| Disease of soft parts . . . | 9 | 8 | 1 | 1 in 9 |
| Compound fracture . . . . . | 6 | 5 | 1 | 1 in 6 |
| Ulcerated and conical stumps . | 4 | 4 | 0 | none. |
| Necrosis . . . . . . . . | 3 | 3 | 0 | none. |
| Tumour of bone . . . . . . | 1 | 1 | 0 | none. |

TABLE IV.—*Ages of* 66 *Cases of Amputation.*

| | No. of Cases. | Cured. | Died. | Prop. of Deaths. |
|---|---|---|---|---|
| From 3 to 10 years . . . . | 6 | 6 | 0 | none. |
| From 11 to 20 years . . . . | 16 | 13 | 3 | 1 in $4\frac{1}{3}$ |
| From 21 to 30 years . . . . | 17 | 15 | 2 | 1 in $7\frac{1}{2}$ |
| From 31 to 40 years . . . . | 10 | 7 | 3 | 1 in $2\frac{1}{3}$ |
| From 41 to 50 years . . . . | 9 | 7 | 2 | 1 in $3\frac{1}{2}$ |
| From 51 to 60 years . . . . | 3 | 3 | 0 | none. |
| From 61 to 70 years . . . . | 4 | 4 | 0 | none. |
| From 75 years . . . . . . . | 1 | 1 | 0 | none. |

The Reporter observes:—In all cases above mentioned, the flap amputation has been preferred as an operation which, it is believed, is not only more quickly performed, and with much less suffering to the patient, but is attended altogether with better results both as regards the form of the stump and the rapidity of its cure. The instrument used for the operation is a straight-backed knife, with an edge gently curving towards the point, and of a length varying with the size of the limb to be removed.

With only one exception, (in which the ordinary tourniquet was applied,) the artery of the limb was commanded by the fingers of an assistant, compression being made with moderate firmness over the axillary or brachial arteries in amputations on the upper extremity; and over the upper part of the femoral artery in operations on the lower limb.

It is found that very little blood is lost when this plan is adopted, because well-directed pressure immediately over the course of the principal artery of the limb completely arrests the flow of blood through that vessel and its offsets, whilst it does not in the least interfere with the return of blood by the veins, which, from their thinner parietes, are the first vessels to be compressed when the tourniquet is applied.

After the removal of the limb also, when the principal arteries have been tied, the smaller vessels are quickly and easily secured by slightly varying the pressure of the finger.

Another advantage derived from this mode of arresting the flow of blood through the limb, is that the operation itself is more conveniently performed than when the tourniquet is used. When both flaps have been cut, they are forcibly retracted by an assistant, whilst by a few sweeps of the knife the bone is denuded for some distance, and sawn through considerably above the point at which the first puncture in the skin was made. Now where a tourniquet is employed, the retraction of the flaps must be interfered with, to a great extent; but where pressure is made by the fingers only, and the rest of the limb is left free, this part of the operation is performed with perfect ease, and the motion of the saw, when applied close against the divided muscle, is not interfered with.

There have been 22 cases of amputation of the thigh—2 primary and 20 secondary. There were 4 deaths.

Nearly all the amputations of the leg were performed close to the tuberosity of the tibia, the stump being left only of sufficient length to rest firmly on the cushion of the wooden leg.

The stump is then completely covered by the dress; and, as it does not project much, it is not so liable to injury, or to become the seat of obstinate and painful ulceration, as is the case with stumps made at the middle of the leg or even a little higher.

Here again the ordinary flap amputation has been had recourse to: a short anterior flap of skin being first made by dividing the skin over the upper end of the tibia in a semicircular form, and then the knife being made to transfix the leg, (at a variable distance behind the tibia and fibula, according to the size and degree of muscularity of the limb,) in order to form a suitable posterior flap.

The portion of muscle thus taken into the posterior flap gives a firmness and roundness to the stump when healed; and does not, in most cases, increase the amount of suppuration, or retard the union of the parts. The only cases in which this mode of amputation is inconvenient, are those in which the patient is muscular and in robust health; as in primary amputations for severe injuries, for example. Under these circumstances the muscles do not appear to have the same power of retraction as in patients who have long been in a low state of health. They are therefore much in the way when the stump is dressed; and by projecting beyond the skin, they prevent its union by the first intention, and occasionally cause some sloughing of the parts.

In two cases where, from the muscularity of the patient, this inconvenience was anticipated, Mr. Liston varied the operation in the following manner.

The anterior flap was made longer than usual, by curving the incision downwards in a semilunar form, and reflecting the skin from the front of the tibia. A posterior flap was then made, also of skin only, and of about the same length as the anterior one. This was reflected from the surface of the gastrocnemius, and the deep structures divided down to the bones, which were separated from the muscles for a short distance before being sawn through.

In these cases the skin flaps healed with unusual rapidity; and the stumps were neat and well covered. In one case, union took place almost entirely by the first intention, and the patient was discharged cured in 25 days after the performance of the operation.

From the accompanying list it would appear that amputation of the leg is an operation which is not so frequently dangerous to life as might be expected. Out of 25 cases, 22 secondary and 3 primary, 22 were successful, giving a proportion of about 1 fatal case out of every 8.

Neither have these generally been found tedious cases; union by the first intention occurring frequently along half and sometimes nearly three-fourths of the line of meeting of the flaps, and the remaining portions granulating without any great amount of suppuration. The average period of time which these patients remained in the hospital before their stumps were completely healed was from forty to fifty days.

*Mode of Dressing the Stump.*—When ligatures have been tied around the principal arteries of a stump, the hæmorrhage from the smaller vessels (which are also tied in cases where the dressing is proceeded with immediately) is arrested by covering the recently-divided surfaces with lint, soaked in cold water.

This is removed and re-applied every few minutes at first, and then at longer intervals, until all bleeding has ceased; and in order to insure the actual application of the cold water to the bleeding surface, the coagula are from time to time gently removed. When the patient begins to recover the shock of the operation, one or two of these smaller arteries perhaps spout out afresh. These, however, if necessary, are easily secured, as the flaps are still separate and exposed: the greater number of vessels, on the contrary, become plugged up with fibrine, and retract within their sheaths.

Thus all chance of disturbance of the dressing, by effusion of blood between the flaps, is prevented; and that without having so many sources of irritation present in the stump, as when many vessels are secured by ligature. Where the patient is unusually nervous and susceptible of pain, tepid water, changed more frequently, may be used in a similar manner.

When all oozing of blood has ceased, and when the divided surfaces become glazed over, (which happens generally in from four to seven hours after the operation,) the wet lint and small remaining coagula are removed, and the dressing of the stump proceeded with. The flaps, which are in the most favourable state for union, are now brought accurately together, and retained by several points of interrupted suture. The number of sutures requisite for this purpose varies from two to four; but more than three are seldom used, even in amputation of the thigh. They are removed frequently in twelve or twenty-four hours; but if the flaps are large and heavy, and the threads cause no redness in the neighbouring skin, they may be left for several hours longer, to prevent any dragging on the recent adhesions. When the flaps are thus in apposition, the edges are more closely brought together by means of strips of plaster applied over the face of the stump, at a little distance from each other, so as to allow of the ready escape of discharge, and the abstraction of the sutures when necessary.

Instead of using, for this purpose, the ordinary resinous plaster, which is a dirty application, readily loosened by discharge, and frequently causing irritation and erythema of the skin, a far more convenient material is found in oiled silk or gold-beater's skin, spread with a solution of isinglass, which is allowed to dry. This plaster is sufficiently firm and tough to support the heaviest flaps; it is very adhesive, and being impervious to water, remains for many days without becoming detached; it does not irritate the skin; and, lastly, as it is quite transparent, the line of union may be seen distinctly through it, and additional support may, at any time, be given to a particular part, where it is seen that the lips of the wound are separating. This dressing is found perfectly sufficient for the first three or four days, or even longer in some cases; the stump being kept gently elevated on cushions covered with oiled silk. No bandage is applied at first, but the stump is left uncovered and cool.

In general, very little inflammatory swelling takes place under these circumstances, and what little does occur is not accompanied with pain, because there is nothing to constrict the parts, and prevent their enlargement.

A bandage is seldom applied before the third or fourth day, though occasionally it is made use of earlier, where the stump is large and heavy, and the union by the first intention not as extensive as usual.

At first, however, the roller is not brought over the face of the stump, but is only allowed to approach the end by circular turns. By this means the discharge is not confined, and the strips of plaster are left undisturbed, these being quite sufficient to prevent the lips of the wound from separating.

When suppuration is fairly established in those parts of the stump which have not united by the first intention, the plaster is usually removed, either entirely or in part, and the end of the stump dressed with lint dipped in tepid water, or in a gently-stimulating lotion, and covered with oiled silk. The bandage also is then brought over the end of the stump in such a manner as to support the flaps together, as the plaster hitherto has done. This simple kind of dressing has the advantage of being cool and clean: and as it may be easily removed, without much pain to the patient, it may be renewed daily.

*Secondary Hæmorrhage.*—In the sixty-six cases here collected, two instances occurred; both in amputation of the thigh. In one case the hæmorrhage proved fatal, as the patient was reduced to an extremely low state, by purulent discharge from the knee-joint, before he consented to amputation. The other patient recovered, after having, first the femoral and then the external iliac artery ligatured. This case is interesting, and may be related.

*Case.*—The patient, a swarthy middle-aged man, was admitted January 28th, 1839, with an immense ulcer over the front of the leg, which had existed for several years, and occasionally bled to a very considerable extent. There was also solid œdema of the lower part of the leg and foot, the skin and cellular tissue being greatly hypertrophied, and the epidermis developed into a kind of horny crust, similar to ichthyosis.

Though the foot was nearly twice its natural size, it was perfectly hard, and did not pit on pressure. The patient was prevented from working, and was beginning to lose his strength and appetite, when he applied, anxious to be relieved of his useless limb.

On the 4th of February he submitted to amputation of the thigh, the ulcer being too extensive to allow of the formation of sound flaps in the leg.

On the 12th of February, eight days after the amputation, secondary hæmorrhage occurred, which was, however, stopped by cold and pressure.

On the *following day* (13th) bleeding recommenced, but with much greater violence. Accordingly a ligature was placed round the femoral artery, just below Poupart's ligament.

On the 15th of February hæmorrhage to the amount of several ounces took place from a small artery (superficial external pudic), wounded in the operation of tying the femoral.

On the 27th of February the ligature came away from the femoral artery, without any bleeding, having been on only fourteen days.

On the 15th of March rapid bleeding occurred from the wound in the groin, which had nearly closed. This was for a time arrested by compression; but in the evening it again returned, and as the patient was excessively weak from a sloughing back, the external iliac artery was tied, as a last resource.

The *following day* several ounces of blood were again lost from the wound in the groin, but this was the last time of its occurrence.

On the 1st of April the ligature separated from the iliac artery, and the wound healed slowly but gradually. The patient after this by degrees recovered his strength and health, and was discharged the 5th of August 1839. He showed himself at the hospital about a twelvemonth afterwards, In excellent health, apparently, and in full work.

An useful statistical contribution.

XII.—COLICA PICTONUM TREATED WITH WARM WATER. By *John Wilson*, M.D., Physician to the Middlesex Hospital.

To relieve the constipation that attends this disorder, it occurred to Dr. Wilson that if an enema were given during the time the patient was in a warm bath, it might then possibly be allowed to pass up the intestinal tube, and be retained so long as to accomplish the object of evacuating the intestines of their morbid secretion, and ultimately of restoring to them their healthy action.

Dr. Wilson relates six cases of constipation from lead, in which this plan was employed. One will be a sufficient sample of the whole.

*Case.*—May 15, 1838.—Matthew Proctor, age forty-five, for thirty years has been a plumber and painter; ill five days, with severe pain coming on in fits over the abdomen, so as to bend him double; has had no evacuation for five days past, though he has had mercury given, to which he attributes the present soreness of his mouth. Afterwards he had five doses of castor oil; yesterday he had three grains of opium in the morning and castor oil in the afternoon, when a mustard poultice was applied over the abdomen; still the bowels persist in the same state as they have been for the last five days. Now, abdomen very hard, but the severity of its pain is mitigated by pressure; tongue white; has had much sickness and frequent vomiting. This is his fourth attack of colic, but he has never had drooping of the wrists.

On admission he was put into a warm bath, and when he had been in it for some time, an elastic injecting tube was given him, with directions to employ it in trying to inject the water of the bath gradually up the intestines, and to persevere, should he feel no pain, nor unpleasant sensation, till he felt a sensation of fullness of the abdomen. In this he succeeded while he continued immersed in the bath; shortly after, and before he quitted the bath, he had an evacuation of lumpy fæces. After leaving it, he was purged four or five times, and relieved from the pain. The next day he had an ounce of castor oil, with ♏ xx. tinct. opii, and a sinapism to the abdomen. The third day the bath and enema while in the bath were repeated; after which, while he remained in the hospital, his bowels never required more than the mistura alba (sulph. mag. ℥ss., carb. mag. gr. v., in mint water), two or three times a-day.

He had no relapse, and was discharged on the 27th.

Dr. Wilson had used the same simple remedy in cases of constipation dependent upon other causes. He relates one. We think that the profession must feel indebted to Dr. Wilson for the hint.

### XIII.—Malposition of the Kidneys; Absence of the Vagina, Uterus, and Fallopian Tubes; Disease of Left Ovary. By *R. Boyd,* M.D., Resident Physician to St. Marylebone Infirmary, and Lecturer on Medicine.

Sarah Richardson, aged 72, died in the St. Marylebone workhouse of chronic disease of the brain and lungs.

The renal capsules were in their usual position, on either side the spine, immediately below the diaphragm. Right kidney situated in the right iliac fossa, below the cæcum, partially concealed by the right ovary, which had a slight peritoneal attachment to it. The renal artery was given off from the right iliac, close to the aorta.

Left kidney in the pelvis below the psoas muscle, resting on the sacrum and origin of the pyriform muscle. An artery which arose from the aorta at its bifurcation, in the situation of the middle sacral, entered the upper end of the kidney; another larger branch from the internal iliac artery, entered the kidney in the usual situation.

Kidneys, ureters, and bladder, in a healthy condition. Right ovary, when divided, presented the natural structure; to its upper or free extremity was attached, by a thin neck, a small oval sac. A round ligament connected the ovary to, and was lost in, the cellular tissue behind the neck of the bladder.

The situation of the left ovary was occupied by a fibrous tumor of an irregular globular shape, connected by a round ligament smaller than that on the right side, but which took a similar course to the bladder.

The Fallopian tubes were not present. There was a slight projection of the peritoneum, behind the bladder, from cellular tissue beneath it. A careful examination of the parts in their recent state was made by Dr. R. Lee, also by Mr. Kiernan, afterwards by Mr. Perry,—no vestige of uterus could be discovered.

The external parts of generation presented no unusual appearance; the mons Veneris but thinly covered with hair: a *cul-de-sac,* about half an inch deep, beneath the orifice of the urethra, is all that exists of vagina.

Mammæ were well developed for so old a person.

As regards the previous history of this women, the only information obtained was, that she had been married, but did not live on amicable terms with her husband.

In the case of Hannah Brown, murdered by Greenacre, the absence of the uterus was observed as in this instance.

### XIV.—PATHOLOGICAL AND SURGICAL OBSERVATIONS ON THE DISEASES OF THE EAR. By *Joseph Toynbee*, Esq.

Mr. Toynbee has been led to believe that deafness must very frequently depend upon a morbid condition of the fibro-mucous membrane lining the cavity of the tympanum. He refers to the observations of Mr. Swan, who relates the particulars of three dissections in which the mucous membrane of the cavity of the tympanum was diseased and thickened, so that the nervous plexus of Jacobson could not be distinguished. One of these cases occurred in an old woman, the second in a man, and the third in a very young woman. In the second case there was also some roughness of the bone. After detailing these appearances Mr. Swan writes—"I believe deafness does not so often depend on a disease of the auditory nerve as has been supposed, but much more frequently on an inflammatory action attacking the membrane lining the tympanum, and involving the small branches of the tympanine nerves." He adds, "although many of the noises may depend on the disordered functions of the auditory nerve, I think they may arise too from these small branches of the glosso-pharyngeal and their communication with the sympathetic in the carotic canal."

Mr. Swan goes on to remark:—

"The consideration of the distribution of the tympanine branch of the glosso-pharyngeal nerve, leads to the conclusion that the tympanum performs more important functions in the production of hearing than have been usually ascribed to it, and that the failure of remedies in cases of deafness which have been termed nervous, may have proceeded very much, not only from the obscure situation of the tympanum, but from the misapplication of the remedies themselves. And I conceive, therefore, as a thickening of the membrane lining the tympanum and involving such delicate nerves, can be so often observed, that many diseases of the ear may be more within the reach of art than has been contemplated, and that by subduing the inflammatory action at its very onset, before the structure of the delicate parts has become so much changed as permanently to impair their functions, many of the worst cases might be prevented." 193.

Mr. Toynbee relates forty-one dissections, of which the following is an abstract.

| | | |
|---|---|---|
| 1 | In a healthy state .. .. .. .. .. .. .. .. .. .. .. | 10 |
| 2 | With simple thickening of the investing membrane .. .. | 6 |
| 3 | With membranous bands proceeding from various parts of the cavity of the tympanum, most frequently connecting the stapes to the circumference of that cavity .. .. .. .. .. | 4 |
| 4 | With slight thickening of the investing membrane, accompanied by the existence of membranous connecting bands.. | 13 |
| 5 | With considerable thickening of the investing membrane and with membranous bands .. .. .. .. .. .. .. .. | 5 |
| 6 | With suppuration of the cavity of the tympanum.. .. .. | 1 |
| 7 | With anchylosis of the base of the stapes to the circumference of the fenestra ovalis .. .. .. .. .. .. .. .. .. | 2 |
| | | 41 |

Mr. Toynbee adds;—" It must appear remarkable that, in thirty-nine specimens of the organ of hearing, taken promiscuously, there should be so large a majority which present appearances indicative of disease. I must observe however, that in several dissections, and more particularly those in which there exist delicate membranous bands, connecting together various portions of the mucous membrane, without the latter being thickened, the deviation from the healthy state is so very slight, that it may be presumed there was not any accompanying derangement of the functions of the organ. The large proportion of specimens which are undoubtedly in a diseased state is very surprising, but it may be less so perhaps when I state that many persons whom I have examined, and who have considered that they *hear perfectly well*, cannot distinguish the ticking of my watch at a distance of two feet and a half, and in some cases, of four or five inches only; though the same watch can be heard distinctly by a healthy ear seven or eight feet from the head. I am thus induced to believe that the function of the ear is impaired much more frequently than is generally supposed; but that such impaired function is not detected without special inquiry. It would be interesting to know whether such derangements are dependent upon the peculiar conditions of the investing membrane of the tympanic cavity, which I have had occasion so frequently to notice in my relation of the above dissections."

" Since the above Paper was read, my attention has been directed to a paper published in the 110th volume of the Philosophical Transactions, entitled, ' On Sounds inaudible by certain Ears, by William Hyde Wollaston, M.D., F.R.S.' The object of the author is to show that there is a very distinct and striking difference between the powers of hearing of different individuals. I am inclined to believe that the deficiency of the power depends upon some pathological condition of the ear, perhaps of a nature similar to that which I have pointed out. Dr. Wollaston states that it never occurred to him to find this defect in any person under twenty years of age—a fact which favours the opinion of its being dependent upon disease or derangement of the organ." 211.

From what we know of Mr. Toynbee, we are satisfied that whatever subject he takes up, he will do ample justice to. We may expect some additions to our knowledge of the pathology of the ear.

### XV.—Two Cases of Dislocation of the Tendon of the Long Head of the Biceps Humeri from its Groove. By *John Soden*, Jun., Esq., Surgeon, Bath.

*Case* 1.—In May, 1839, J. Cooper was engaged in nailing down a carpet, when, on rising hastily from his occupation, his feet slipped, and he fell backwards on the floor; in order to break the force of his fall, he involuntarily placed his arm behind him, and, by so doing, received the whole weight of his body upon the right elbow; that joint, however, though the only part struck, received no injury, for the shock was instantly transmitted to the shoulder, and there the whole effects of the accident were sustained.

Acute pain was immediately experienced, and the man supposed that he had suffered either a fracture or dislocation, but, finding that he could raise the arm over his head, he felt re-assured, and endeavoured to resume his work. The pain, however, compelled him to desist, and he went home. When Mr. Soden saw him on the following morning, the joint was greatly swollen, tender to the touch, and painful on very slight motion; there was then no possibility of his placing his arm over his head, as he said he had done immediately after the accident. Mr. S. thought the case one of severe sprain. Unusually active means were required to subdue the inflammation, and, at the end of three weeks, though the swelling was much reduced, the tenderness in front of the joint, and pain on certain movements of the limb, were scarcely less than on the day after the occurrence of the accident.

On comparing the joint with its fellow, now that the swelling had subsided, a marked difference was observable between their respective outlines; the injured shoulder was evidently out of drawing, but without presenting any glaring deformity. When the man stood erect, with his arms dependent, the distinction was very manifest, but difficult to define: there was a slight flattening on the outer and posterior parts of the joint, and the head of the bone looked as though it were drawn up higher in the glenoid cavity than it should be. Examination verified this appearance in two ways: 1st, on moving the limb, with one hand placed upon the shoulder, a crepitating sensation was experienced under the fingers, simulating a fracture, but, in reality, caused by the friction of the head of the humerus against the under surface of the acromion; 2ndly, on attempting abduction, you found that the arm could not be raised beyond a very acute angle with the body, from the upper edge of the greater tubercle coming in contact with that of the acromion, and thus forming an obstacle to all further progress. The head of the bone was also unduly prominent in front, almost to the amount of a partial dislocation.

For all useful purposes the arm was powerless—the man was unable to raise the smallest weights from the ground, on account of the severe pain induced by any exercise of the biceps muscle; otherwise, the underhand motions were not limited, the arm could be readily swung backwards and forwards, and the patient could grasp an object firmly, and without pain, so long as he made no attempt to raise it. The locking of the humerus and acromion on abduction, in the manner before alluded to, of course formed an insuperable opposition to all the overhand motions.

The pain caused by the action of the biceps was described as very acute, and extending through the whole course of the muscle, but felt chiefly at its extremities, the lower equally with the upper; when not excited by muscular action, it was referred to the front of the joint, and confined to the space between the coracoid process and the head of the humerus, which spot was marked by extreme tenderness and some puffy swelling.

The patient being of a rheumatic habit, inflammatory action of that character was soon established in the joints, so that the peculiar symptoms of the injury were masked by those of general articular inflammation, which added greatly to the man's sufferings, and materially augmented the difficulty of the diagnosis.

On Nov. 9, 1839, the patient met with a compound fracture of the skull, and died.

On examining the joints, the accident was found to be a dislocation of the long head of the biceps from its groove, unaccompanied by any other injury. The tendon was entire, and lying enclosed in its sheath on the lesser tubercle of the humerus; the capsule was but slightly ruptured; the joints exhibited extensive traces of inflammation; the synovial membrane was vascular and coated with lymph; recent adhesions were stretched between different parts of its surface, and ulceration had commenced on the cartilage covering the humerus, where it came in contact with the under surface of the acromion; the capsule was thickened and adherent, and in time probably anchylosis of the joint would have taken place.

Mr. Soden thinks that the biceps may act as an antagonist to those muscles of the shoulder-joint, which tend to pull the humerus up. With this consideration, he says, of the tendon of the biceps in its capacity of a capsular muscle, we can understand why, when the tendon is ruptured or displaced, the head of the bone should rise upwards and forwards,—a precisely opposite direction to that in which the tendon would, when *in situ,* tend to direct it.

*Case* 2.—William Mountford, ætat. 55, was admitted into the Bath United Hospital, on the 24th of April last, having been severely injured by a quantity of earth falling upon him. He had sustained, in addition to some severe contusions, a dislocation forwards of the humerus, and fractures of some of the ribs on the same side. The man lingered for a few days, and died from hæmorrhage into the cavity of the chest, in consequence of the lung having been perforated by a fractured rib.

Unusual difficulty had been experienced in the reduction of the dislocation, which was very high up, but it had been at last effected.

On examining the joint, a rent was discovered in the capsule on its inner side, through which the head of the bone had passed; the sheath was torn up, and the tendon having escaped, had slipped completely over the heads of the bone, and was lying at the inner and posterior part of the joint.

I consider that the difficulty of reduction was attributable to the complication of the injury of the biceps, for the inferences from the former case would lead us to expect that, had the tendon been *in situ,* it would have aided the return of the bone; but its influence being removed, the resistance of the upper capsular muscles became doubled, and twice the amount of force was consequently required to overcome it. This may be considered as a rule applicable to all dislocations forwards, where the head of the bone is not thrown *below* its original level.

### XVI.—An Account of two Cases of Aneurism of the Superior Mesenteric Artery, in one of which Jaundice was induced by Pressure of the Sac. By *James Arthur Wilson,* M.D. Physician to St. George's Hospital.

*Case.*—Ann Pinchin, widow, aged 24, admitted under Dr. Wilson's care, Feb. 24, of this year. She had been ill four months, and her general appearance was that of great depression and exhaustion. The case in its progress presented the usual symptoms of jaundice in a very aggravated degree; it was very little influenced by the means employed for its relief, and was remarkable principally for the severity of the pain complained of between the shoulders, along the track of the six or eight lower dorsal vertebræ. There was also occasional pain in the epigastrium and right hypochondrium,* both of which regions were carefully examined from time to time, without any information being thus obtained as to the immediate cause of the disease.

There was great dejection of mind, with entire loss of appetite and want of muscular power; the skin became more intensely coloured as the case advanced to its termination, and the saliva voided at this period of the disease stained the linen, on which it was received, of a deep yellow colour; there was also distinct evidence of the same tint in the menstrual flux, which occurred twice during the seven weeks that the patient passed in the Hospital. She died April 12, in a state of great general exhaustion, much aggravated by a mercurial salivation, following the administration of some small doses of calomel and opium.

The body was examined April 13th, twenty-four hours after death.

On removing the integuments, a stain of yellow was observed generally throughout the fat, and the exposed inner structures of the body. In the duodenal region, on raising the liver from the subjacent viscera, a large globular tumor was seen extending itself from behind the head of the pancreas upwards, forwards, and outwards, to the right side of the body, in the direction of the ductus communis choledochus, so as to occupy the greater part of the space usually defined by the laminæ of the small omentum. The tumor was smooth on its surface, of firm texture, and was found to be the sac of an aneurism, situated in the trunk of the superior mesenteric artery, commencing about an inch from its origin, and extending itself in the directions mentioned.

The ductus communis was in close and prolonged contact with the walls of the sac, by which it was compressed in its whole extent: it was, however, pervious to the probe, and bile could readily be squeezed from its orifice into the duodenum.

The liver was of a dark livid tint, but was healthy in its general structure; its pori biliarii were universally enlarged and greatly distended with bile, so that the diameter of many of these vessels exceeded that of the larger gall ducts in ordinary cases. The gall-bladder contained a large qnantity of healthy bile, with a few small gall-stones.

Dr. Wilson very judiciously observes:—

"Although the instance I have described of jaundice depending on aneurism of a large branch of the aorta be a solitary one, yet, by the recollection of it, in protracted and intractable cases of the disease, we may be the more impressed with the necessity for making close and frequent examination of the upper region of the abdomen, by the *ear*, as well as by the eye and the hand, during the continuance of the symptoms.

It may likewise teach us caution in our estimate of the probable duration of cases of this disease, and in our conjectures as to the mode of their termination.

If jaundice be occasionally the effect, simple and direct, of pressure on the

---

* The pain was very severe, and returned in paroxysms with marked intervals of suspension.

gall-ducts by an aneurism of a large branch of the abdominal aorta, then, in the treatment of cases classed under this name of jaundice, it will be well to remember that they may sometimes close with the awful suddenness of hæmorrhage from the main trunk of the circulation.

For such possible termination of a disease, chronic in its general character, and not usually considered as dangerous, the friends of the patient, in all cases warranting suspicion, should surely be prepared.

I am, moreover, willing to hope, that the relation of this case may tend to establish one caution the more against the mischievous routine of practice, now happily less frequent among reflecting physicians, of administering mercury in all cases of supposed 'liver affection,' and of jaundice, as included among such 'affections.'" 225.

*Case* 2.—William Frost, coachman, aged 42, admitted Feb. 11, 1835, complaining principally of a tumor pulsating in the epigastric region. It was of the size of a small orange, and, as he lay flat on his back, was observed to project rather to the left of the scrobiculus cordis. It was painful on pressure, and was moveable in nearly every direction, but most easily so towards the left side,

When the patient turned on this side, the tumor "fell at once under the ribs," and could no longer be felt.

On his turning to the *right* side, the tumor fell over in the same direction, and could still be distinctly felt in the front and to the right of the epigastric region.

Two or three months before his admission, he had suffered much from shortness of breath, with pain in the loins, and "between the shoulders," along the lower dorsal vertebræ. In a fortnight after his admission, he became very costive, and was attacked by cough, with profuse hæmoptysis, for the first time since his illness. From this time until the death of the patient on July 10th in the same year, large quantities of blood were frequently brought up by cough, and latterly by vomiting. The blood drawn from the arm subsequently to these attacks, and for the relief of other symptoms, was always more or less cupped and buffed; the pulse was never irregular, and generally of moderate frequency. As the case advanced, the costiveness became obstinate, the appetite failed, the pain between the shoulders, along the lower dorsal vertebræ, became more severe, and there was much occasional suffering from cramps in the legs, with numbness and tingling in them, as in the arms and hands.

The tumor became more and more tender to the touch, and, some weeks before death, was observed to have changed its position from the *left* to the *right* side of the epigastrium.

Towards its close, the case presented many phthisical symptoms, and under these the patient gradually sank.

On examination after death, the aneurismal sac in this case, as in the other, was found to be situated in the trunk of the superior mesenteric artery. It was large, kidney-shaped, extending *upwards*, *forwards*, and *outwards*, to the right side of the body, thus raising with it the pancreas, which viscus lay on the upper boundary of the tumor.

The walls of the sac, especially in front, were firm and thick, and were enveloped by a transparent layer of peritoneum. The sac communicated directly with the aorta, by a long and wide opening with a smooth edge.

It contained many coagula; those next to the aorta were loose and black, in the front of the sac; they were very firm in texture, much laminated, and of a gray colour.

The larger branches of the superior mesenteric artery were distinctly recognized at the projecting end of the sac: they were open, and pervious to the blow-pipe, which passed readily, from them, through the loose coagula of the sac into the aorta.

The cæliac and superior mesenteric arteries were given off as usual from the aorta, and, with it, were healthy in their structure. The lungs were extensively diseased by vomicæ, and by tubercles of the common kind.

Dr. Wilson remarks:—

"It may be observed, that the two cases, which I have now described, of aneurism of the trunk of the superior mesenteric artery, do not present many symptoms in common: they were respectively distinguished during life, the one by jaundice, the other by vomiting of blood.

Both cases were remarkable for the severity and constancy of the pain in the *middle* of the back, referred by both patients to 'between the shoulders.'

The difference in the leading symptoms of the two cases to which I have alluded, finds its explanation in the difference of the situation of the aneurismal sac in relation to the pancreas, the liver, and the surrounding structures." 230.

### XVII.—ON CONGENITAL TUMORS OF THE PELVIS. By *Edward Stanley*, F.R.S., Surgeon to St. Bartholomew's Hospital.

Mr. Stanley observes that there are various forms of congenital tumor attached to the pelvis, the discrimination of which becomes of much importance with reference to the question of their removal by operation.

In 1836, Mr. Stanley was requested to see a child, four months old, born with a soft pendulous tumor, about the size of an orange, attached to the lower and back part of its body. In every other respect the child appeared to be perfectly formed and healthy. With the growth of the child, the tumor progressively increased, and in proportion to the rest of its body; it continued to thrive well to the age of two years, when it was attacked by measles, with other children of the family, producing much constitutional derangement, under which it gradually sunk. At the time of its death, the following were the dimensions and general characters of the tumor: its circumference measured fourteen inches and a half; a line extending from its base to the most prominent point of its centre measured four inches and a half. The base being very broad, covered the whole of one buttock, and extended across the sacrum to the opposite side of the pelvis. The skin covering the tumor was natural; some large and tortuous veins were seen ramifying in the subjacent cellular tissue. Upon some parts of the tumor there were shallow grooves, which were supposed by many who examined the case to correspond with depressions between folds of intestine within the tumor. The surface of the tumor was generally soft, but in some situations, portions of a firm substance, resembling isolated pieces of cartilage, were recognised in it, and it was remarked that these points of resistance were not always to be felt in the same situation. Pressure of the tumor did not cause a diminution of its size, so as to justify the belief that any portion of it receded into the body. A finger passed into the rectum discovered a portion of the tumor extending into the cavity of the pelvis by the side of the intestine. When the child cried loudly, the tumor became tense; this showed its communication with the interior of the body, and it was thought by some to indicate the probability of its communication with the spinal canal, in accordance with the views of Magendie, that there occurs a movement of the contents of the spinal canal in connexion with respiration, and dependant on distention of the vessels of the cord and its membranes.

Many surgeons had seen the case. Mr. Blizard alone thought the removal of the tumor practicable.

A cast of the tumor was first taken, and then its interior was examined. This was found to consist of an assemblage of different tissues. One portion of it was solid throughout, and closely resembling in its characters the ordinary fibrous tumor of the uterus. Another and larger portion consisted of two cysts, one enclosing the other; the sides of these cysts were membranous, and their texture dense and fibrous, each cyst containing a transparent yellow fluid. A narrow and solid portion of the tumor was found to extend through the inferior aperture of the pelvis upwards within its cavity, nearly to the top of the sacrum, chiefly occupying the right side of the pelvis, and in consequence compressing the bladder and rectum, not, however, apparently to the extent of materially interfering with their functions. There was no attachment of the tumor to the sacrum, otherwise than by loose cellular tissue. The sacral canal was completely closed, and accordingly had no communication with the tumor. The information derived from the examination of the extent and connexion of the tumor appeared to confirm the opinion that its removal might have been safely undertaken in an early stage, when, from the smaller extent of the tumor, it might have been practicable to draw downwards the portion of it from within the pelvis, as it had no other connexions with the surrounding organs than by loose cellular tissue.

Mr. Stanley introduces several cases for which we must refer to the Transactions themselves. His inferences from them are deserving of notice. He says that the cases on record admit of being arranged in four classes.

"First. The cases wherein the tumor is composed wholly of morbid structures, which, although formed during fœtal life, have no peculiar character, the solid tissues mostly resembling the ordinary fibrous tumors of the uterus, and the membranous cysts being analogous in their nature and contents to the corresponding anormal structures formed at other periods of life.

Secondly. The cases wherein the tumor is composed of morbid structures in conjunction with isolated portions of perfectly-formed animal organs, having no other relation to the living being with which they are connected, than as they are dependent upon it for the means of nutrition and growth. These cases must be considered to belong to the class of parasitic monsters, constituting intra-fœtation, the inclusion of one fœtus within another, and, in accordance with the present theories on this subject, supposed to result from the cohesion or intus-susception of germs, when more than one ovulum is contained in the same vesicle, under which circumstances there will arise either the union of two perfect fœtuses, as in the instance of the Siamese twins, or the growth of one fœtus to its perfect form, with but the portion of another fœtus attached to it, as in the remarkable case recorded by Velpeau, where a tumor which was removed from the scrotum of an adult was found to consist of several bones, with other distinct parts of a fœtus.

Thirdly. The cases wherein the tumor, being of the nature of spina bifida, consists of a membranous cyst, communicating with the interior of the theca vertebralis.

Fourthly. The cases wherein the tumor is composed either wholly, or in part, of membranous cysts, communicating with the spinal canal, but exteriorly to the theca; thus in one of the cases related, a probe passed from the cyst, of which the tumor in part consisted, through one of the anterior sacral holes into the cellular tissue between the theca of the cord and the bony walls of the canal.

It will be observed, that in all the cases noticed in this paper, the congenital tumor projected from the posterior and inferior part of the pelvis; its situation, consequently, was such as to allow of removal by operation but from the apprehension that there might be some deeper portion of it extending to the interior of the pelvis, and more especially from the apprehension that the tumor might be of the nature of spina bifida, and accordingly that its interior would be found to be continuous with the membranes of the spinal cord.

We learn from the foregoing histories that the general character of these congenital tumors, whatever may be their nature, is to increase progressively, and in proportion to the rest of the body; hence arises the important question of their removal: and it must be added, that this question will in general be extremely difficult of decision, for the reason that no outward mark or symptom can be referred to as distinguishing the tumor composed wholly of morbid products, and having no other connexion with the body of the child than by cellular tissue, from the tumor which, by the continuity of its interior with the membranes of the spinal cord, is of the nature of spina bifida; yet the operation of removal, in one case accomplished with a fair prospect of success, would, in the other, be certainly fatal. And, accordingly, it has happened in one of these cases, that an operation commenced with the expectation of a successful result, has been stopped in its progress by the discovery of a pedicle extending from the tumor to the interior of the spinal canal. Mobility of the congenital tumor does but indicate the probability of its having no connexion with the vertebral canal, as the means of this connexion may be a narrow pedicle, permitting free movement of the tumor upon the walls of the pelvis. Also to the existence of a narrow pedicle and a small opening of communication with the vertebral canal, we may refer for explanation of the frequently observed fact of pressure of the tumor causing no portion of its contents to recede into the canal, and thus occasioning symptoms of compression of the spinal cord or brain. Any derangement of the nervous functions in the lower limbs would of course be evidence of the probability of the connexion of the tumor with the spinal cord; but it must be recollected that the instances are not infrequent of spina bifida co-existing with a perfect integrity of function in the spinal cord and its nerves.

A circumstance of physiological interest will be noticed in two of the foregoing cases, namely, the existence of a fluid in the isolated portion of intestine within the parasitic monster, which in colour and other obvious characters closely resembled meconium, although there existed no liver or other distinct hepatic apparatus which could have furnished the colouring matter of this fluid, and there was certainly no communication between this portion of intestine and the intestinal canal of the child to which the parasitic monster was attached. An analogous fact occurred to my observation, many years ago, in the examination of an acephalous lamb, in which, with perfectly-formed stomach, intestines, spleen and kidnies, the liver was wholly wanting, and yet within the intestines, especially, the large, there was found a considerable quantity of a dark yellow and thick fluid, not to be distinguished by its appearance from meconium. When diluted, the colour of this fluid was exactly that of healthy bile, but it was not bitter to the taste, and in this respect it differed from the perfect meconium of the human fœtus, which imparts to the tip of the tongue the peculiar bitter flavour of bile." 244.

An instructive communication.

This completes our account, itself complete, of the volume before us. A volume fraught with practical information, and reflecting honour on the Society and the Profession.

